# STGIC: A graph and image convolution-based method for spatial transcriptomic clustering

**DOI:** 10.1371/journal.pcbi.1011935

**Published:** 2024-02-28

**Authors:** Chen Zhang, Junhui Gao, Hong-Yu Chen, Lingxin Kong, Guangshuo Cao, Xiangyu Guo, Wei Liu, Bin Ren, Dong-Qing Wei

**Affiliations:** 1 School of Life Sciences and Biotechnology, Shanghai Jiao Tong University, Shanghai, China; 2 Department of Computer Science and Engineering, Shanghai Jiao Tong University, Shanghai, China; 3 College of Life Science and Technology, Huazhong University of Science and Technology, Wuhan, China; 4 State Key Laboratory of Public Big Data, College of Computer Science and Technology, Guizhou University, Guiyang; 5 Smart-Health Initiative, King Abdullah University of Science and Technology, Jeddah, Saudi Arabia; 6 Marine Science and Technology College, Zhejiang Ocean University, Zhoushan, China; University of Pittsburgh, UNITED STATES

## Abstract

Spatial transcriptomic (ST) clustering employs spatial and transcription information to group spots spatially coherent and transcriptionally similar together into the same spatial domain. Graph convolution network (GCN) and graph attention network (GAT), fed with spatial coordinates derived adjacency and transcription profile derived feature matrix are often used to solve the problem. Our proposed method STGIC (**s**patial **t**ranscriptomic clustering with **g**raph and **i**mage **c**onvolution) is designed for techniques with regular lattices on chips. It utilizes an adaptive graph convolution (AGC) to get high quality pseudo-labels and then resorts to dilated convolution framework (DCF) for virtual image converted from gene expression information and spatial coordinates of spots. The dilation rates and kernel sizes are set appropriately and updating of weight values in the kernels is made to be subject to the spatial distance from the position of corresponding elements to kernel centers so that feature extraction of each spot is better guided by spatial distance to neighbor spots. Self-supervision realized by Kullback–Leibler (KL) divergence, spatial continuity loss and cross entropy calculated among spots with high confidence pseudo-labels make up the training objective of DCF. STGIC attains state-of-the-art (SOTA) clustering performance on the benchmark dataset of 10x Visium human dorsolateral prefrontal cortex (DLPFC). Besides, it’s capable of depicting fine structures of other tissues from other species as well as guiding the identification of marker genes. Also, STGIC is expandable to Stereo-seq data with high spatial resolution.

## Author summary

Spatial transcriptomics detect gene transcription profile with high spatial resolution even to sub-cellular level, which is very helpful to characterize organization and architecture of tissues and find marker genes corresponding to sub-structures. Therefore, it is much more informative than traditional transcriptomics for interpreting the biological processes underlying tissue development and disease progression. Clustering to clarify each spatial domain of tissues in which cells present similar transcription profile and histology is a primary task for analyzing spatial transcriptomics data. To solve the important problem, we propose the method STGIC combining deep learning skills in both graph and image, since we notice that image methods tend to display good performances on some samples in benchmark datasets which existing graph methods are not good at, and vice versa. Besides, we substitute the vanilla form of graph convolution frequently used in this field with the one which can find the optimal order of neighbor involved in feature aggregation adaptively for different graphs. STGIC displays high clustering performance and identifies spatial domains which can be used to depict fine-grained structure and help ascertain marker genes of different tissues from different species sequenced with 10x Visum and Stereo-seq techniques.

## Introduction

Single-cell transcriptomics (SC) technique has been fully developed to promote the research of a myriad of fields in life science via its advantage of high resolution to make clear the transcriptomic profile in every single cell [1]. SC clustering requires grouping cells of the same type together with only gene transcription information [2]. Methods originally for community detection have been used for SC clustering, such as Leiden and Louvain [3,4]. Spatial transcriptomic (ST) technique has also been rapidly developed in recent years. Compared with SC, it takes account of one more aspect of the spatial information of the sequencing unit, also referred to as a spot [5]. Spatial resolution of most existing ST technique is generally not so high as SC and the improvement of spatial resolution is usually realized at the cost of drop in the number of different kinds of transcripts captured in one spot, which is termed as gene-capturing efficiency hereafter. At present, 10x Visium [6,7], Slide-seq [8,9] and seqFISH [10] have been recognized as the most popular technique for ST sequencing. The latter two are both in-situ hybridization based techniques which are featured by irregular lattices on chips. Slide-seq boasts higher spatial resolution of 10 μm to attain nearly cellular level than 10x Visium with the spatial resolution of 55 μm. seqFish likewise achieves high spatial resolution but somewhat low gene-capturing efficiency. Despite the inferiority of 10x Visium in terms of spatial resolution, it does much better in the gene-capturing efficiency of each spot for the mentioned paradox between spatial resolution and gene-capturing efficiency. Stereo-seq [11] is another technique with even higher spatial resolution than Slide-seq to achieve sub-cellular level, and also the gene-capturing efficiency is still open to improve. Both 10x Visium and Stereo-seq have their spots arranged into regular lattices on the chips, as are contrast to in-situ hybridization based techniques.

ST clustering aims to divide all spots from a sample slice into spatial domains according to both spatial vicinity and gene transcription similarity. Each spatial domain consists of continuously distributed spots, all of which present relative coherence in transcription profile. Based on division of spatial domains, many important downstream works can be done such as detecting spatially variable genes (SVGs) whose expression distribution have significant correlation with their spatial locations. SVGs are often markers for specific structural components of tissues or function as regulator for signal transduction pathways [12]. Therefore, identification of SVGs is tremendously helpful to unearth the underlying biological mechanisms behind tissue development [13], onset and progression of diseases [14].

So far, ST datasets with high quality of cluster or cell type labels have still been very scarce since the experimental labeling process is terribly time-consuming and costly. The 10x Visium human DLPFC dataset [15] composed of 12 samples and owning generally acceptable annotated labels about spatial domains has enabled the test of various ST clustering algorithms about clustering performance. Hence, the DLPFC dataset has been taken as benchmark to measure the precision of cluster assignment generated by related algorithm.

At the beginning of the development of ST techniques, Leiden and Louvain considering only gene transcription information had also been used frequently for ST clustering despite the disregarding of the spatial information. The situation was then changed by the application of graph deep learning to this field. SpaGCN [16] in late 2021 which adopts GCN [17] with an adjacency constructed according to spots’ spatial distances to extract features from information on both gene transcription and histological image. The method can be used for clustering and detecting SVGs. Shortly after SpaGCN, STAGATE [18] was reported as a comprehensive ST toolkit, which carries out spots clustering via GAT and auto-encoder framework [19,20] to reconstruct the gene expression information, the spatial coordinates is used to identify neighborhood for each spot, but different from SpaGCN, it doesn’t take cues from histological image. DeepST [21] reported in late 2022 takes advantage of a pre-trained neural network to extract feature from histological image and integrate it with gene expression information to construct a feature matrix. Moreover, graph neural network auto-encoder and denoising auto-encoder are simultaneously used to extract the latent feature which is further taken by Leiden to generate clustering labels. The foregoing graph learning methods are based on single graph, however, STMGCN [22] goes beyond the custom by constructing two graphs from one sample. Besides the adjacency calculated as SpaGCN does, another adjacency with binary value 0 and 1 is derived from cosine similarity calculated with spatial coordinates and only the weights of the elements corresponding to the top 20 neighbors in the adjacency are conferred to the value 1. Attention mechanisms are used to fuse the embeddings from the two graphs to obtain the final embeddings for clustering.

Besides graph-based deep learning method, an image convolution-based method abbreviated as TESLA [23] is also developed SpaGCN’s author to annotate cell types *in situ* on histological images for tumor tissues. TESLA is fed with a 2-channel image with one channel derived from the gray degree converted from histological image and another from the transcription information of marker genes of all spots. The input is processed by several blocks containing convolution layers with kernel size of 3×3, the output of these blocks is used to generate pseudo-labels to supervise itself with cross-entropy loss and the spatial continuity is ensured by the constraint that longitudinally and transversely neighbored pixels should be similar in embedding space. The main configuration of TESLA refers to an unsupervised image segmentation method [24], rendering TESLA the first trial of image-based deep learning method in ST, though not for identifying spatial domains. Noticeably the input image is not simply histological images, rather it is virtualized from gene expression information. ScribbleDom [25] is another image-based method employing the convolution framework of Inception to extract feature from the virtual image. It depends on expert’s annotated histological images to raise clustering accuracy by a large extent from the level attained by its unsupervised training which is initiated by mclust generated pseudo-labels.

In addition to deep learning methods, Bayesian statistic methods also find their way in ST analysis among which BayesSpace [26] are the first reported paradigm and has made itself a representative of the application of Bayesian statistics to ST clustering.

Herein, we introduce STGIC to deal with ST clustering problem specifically for techniques adopting regular lattices on chips, such as 10x Visium and Stereo-seq. STGIC consists of AGC [27] for pre-clustering and DCF for clustering. AGC is quite different from common graph convolution mainly in terms of two aspects, on one hand, it doesn’t depend on any trainable parameters, on the other hand, it works adaptively to detect the appropriate order of neighbors to aggregate for different graphs and is thus more likely to avoid over-smoothing and under-smoothing spots’ embeddings to achieve a good clustering performance. AGC functions to provide high quality of pseudo-labels for pre-training of DCF. Unlike the convolution framework adopted in TESLA, DCF adopts different combinations of convolution kernels for 10x Visium and Stereo-seq. Special steps have been taken to ensure that feature extraction of every spot-corresponding pixel pays attention to only the neighboring spot-corresponding pixels within a certain spatial distance and neglect all those corresponding to no spots or far away, besides, the extent to which neighbors are paid attention to by a spot is the same among those equally distant from the spot. Training of DCF doesn’t either mechanically follow what is adopted in the foregoing unsupervised image segmentation algorithm, as is embodied not only by the AGC assisted pre-training, but also the transferring of a self-supervision loss originally designed for embeddings generated by GCN to that extracted from the feature image output by DCF, more directions to compute spatial continuity and more strict restraints on the eligibility of spots being involved in calculating cross entropy.

Tests of STGIC with the 10x Visium DLPFC benchmark and other datasets of different tissues, species and sequencing techniques indicate the following points: (*i*) STGIC attains SOTA clustering performance on the benchmark with high mean and median adjusted Rand index (ARI). (*ii*) STGIC can delineate the structure of human and mouse brains and human breast cancers in fine-grained scale. (*iii*) STGIC is competent for 10x Visum and Stereo-seq owning regular lattices.

## Results

### Pipeline of STGIC to analyze the benchmark of 10x Visium DLPFC

**Fig 1:**
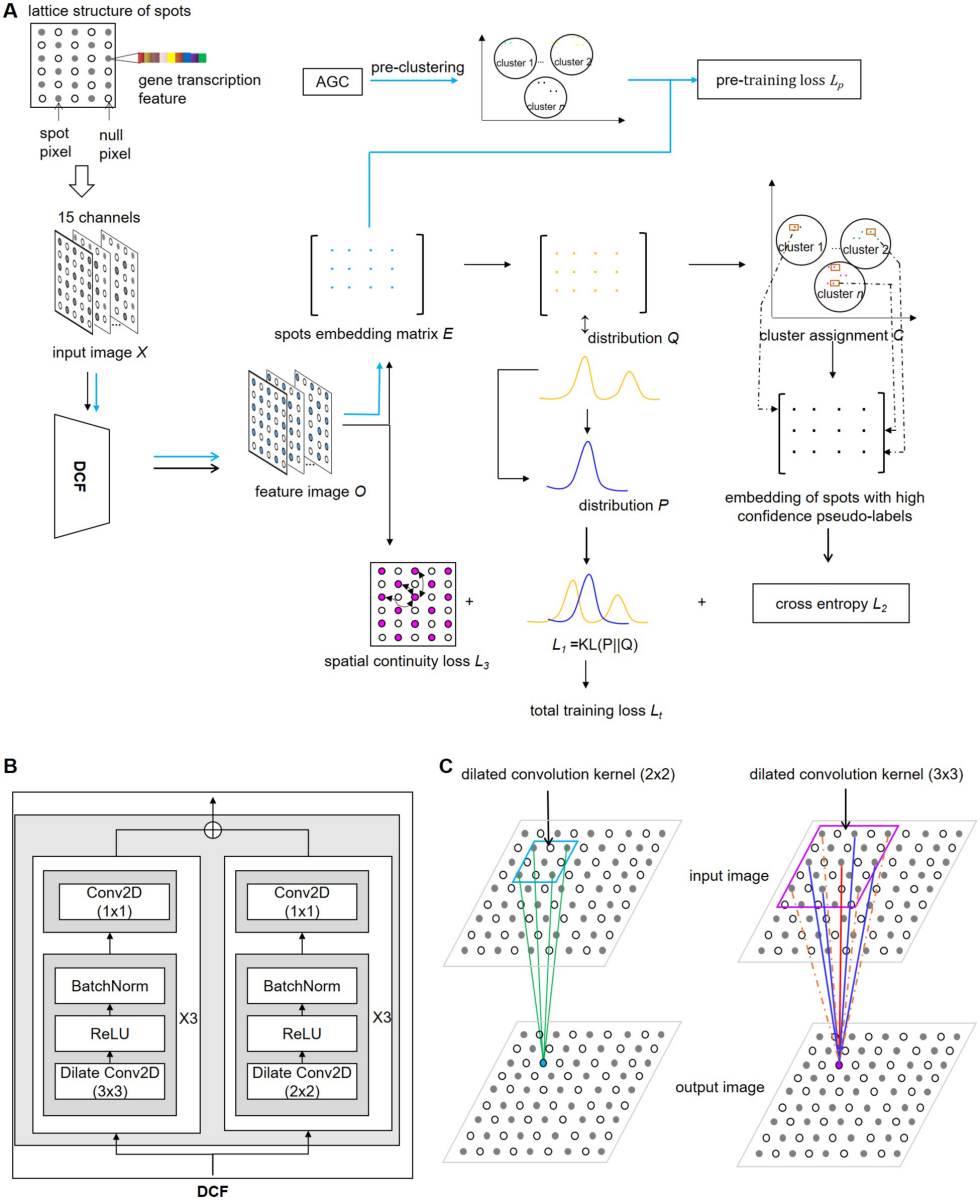
Overview of STGIC. A. Pre-training and training of DCF. Steps of pertaining is linked by blue arrows, while those of training by black arrows. A virtual image is converted from the lattice of 10x Visium and gene expression information to serve as the input of DCF. AGC works to generate a cluster assignment *C*_*0*_ for supervising the pre-training of DCF. Training of DCF starts with the trainable parameters initialized by values identified during the pre-training stage and is carried out with objectives of minimizing the total loss consisting of three components. B. The detailed structure of DCF for 10x Visium. DCF is composed of two sub-frameworks depending on convolution kernels with dilation rate of 2 and size respectively of 2×2 and 3×3. C. Two kinds of Convolution kernels are used in DCF. Both have dilation rate of 2, but one has kernel size of 2×2 and the other has that of 3×3. Convolution kernel shares the same weight at positions with equal distance to the center. Lines with the same color represent the same kernel weight when extracting feature from a receptive field. Orange dash lines represent the weight of zero to ignore these spots during feature extraction.

Implementation of STGIC is divided into stages, including preparation of input for AGC and DCF, pre-clustering with AGC, pre-training and training of DCF (Fig 1).

Data of each sample are stored in a h5ad file from which a gene transcription matrix *M*_*0*_ *∈ R*^*s0*×*g0*^ is extracted, *s*_*0*_ represents number of spots and *g*_*0*_ represents number of genes in unfiltered data. The matrix records unique molecular identifier (UMI) counts of genes detected in all spots. Besides, two kinds of coordinates are contained in 10x Visium data to identify positions of spot-corresponding pixels in the histological image and to index spots in the lattice, the two kinds of coordinates are termed as pixel coordinates and lattice coordinates henceforth. Genes expressed in fewer than three spots are filtered. The counts of gene expression of each spot is normalized by being divided with total UMI counts of all genes in the spot, multiplying 10,000 and taking natural logarithm [16]. The resulting normalized gene expression matrix is denoted as *M* ∈ *R*^*s*×*g*^, *s* and *g* respectively represents the number of spots and that of genes in filtered data. The ST clustering task is to assign a cluster label to each spot and the cluster number *n* is usually pre-specified.

Pixel coordinates are used to compute the distances between each pair of spots whereby to construct a distance matrix *D* ∈ *R*^*s*×*s*^, based on which the adjacency *A* ∈ *R*^*s*×*s*^ is computed with Gaussian kernel and further a symmetrically normalized Laplacian matrix *L* ∈ *R*^*s*×*s*^ is computed. The top 50 principal components (PCs) are extracted via principal components analysis (PCA) of the matrix *M* to get the feature matrix *F* ∈ *R*^*s*×50^.

**Fig 2:**
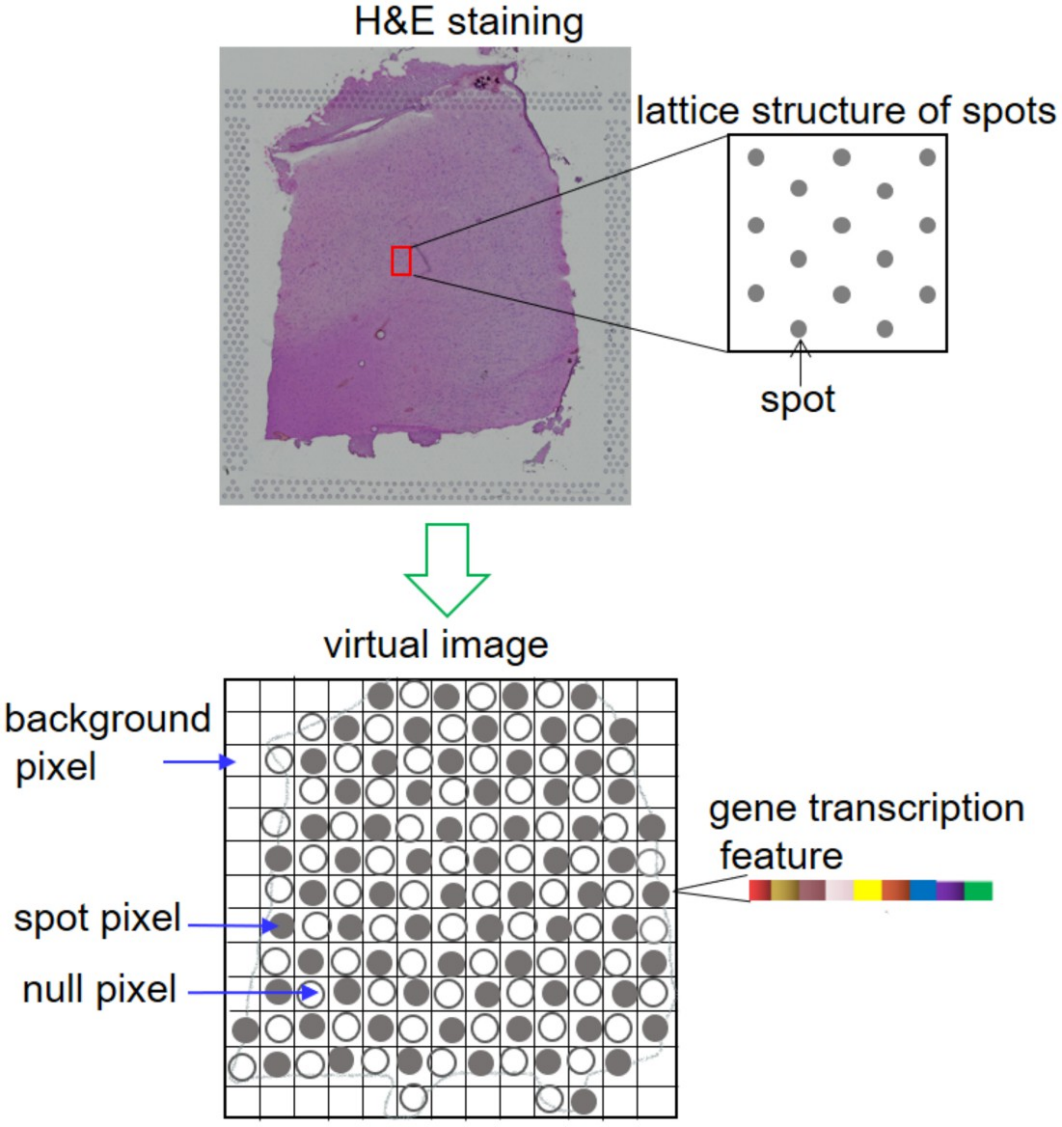
Lattice structure of 10x Visium and the virtual image based on it. Spots are arranged alternately on a chip to form a regular array. Pixels of the virtual image are denoted with small squares. Spot pixels are denoted with squares containing solid circles. Null pixels are denoted with squares containing hollow circles. Background pixels are denoted by squares not containing any circle. The pixel values of spot pixels are derived from the transcription information of spots.

A virtual image *X* ∈ *R*^15×*h*×*w*^, is constructed with lattice coordinates and gene transcription information as the input for DCF, the channel number is 15, *h* and *w* represent the height and width of the image. For 10x Visium lattice, spots in successive rows are staggered by one spot (Fig 2). To generate an integral image, each staggering location is filled with a pixel which doesn’t correspond to any spot and is termed as null pixel while pixels corresponding to spots on the lattice are termed as spot pixels. Pixels whose corresponding locations on the lattice are outside the area of tissue are termed as background pixels. The pixel values of spot pixels are derived by taking the top 15 PCs generated by PCA with the matrix *M* and those of null and background pixels are also imputed.

**Fig 3:**
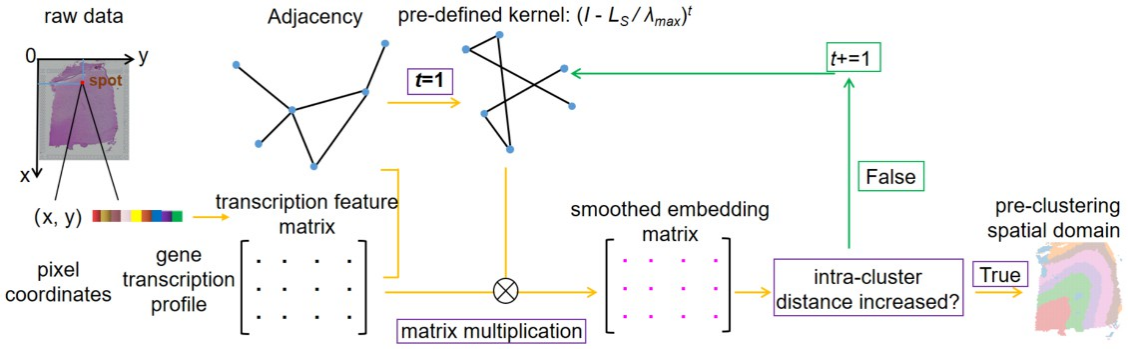
Workflow of the pre-clustering method AGC in STGIC. Pixel coordinates of spots is used to compute the Euclidean distance based on which adjacency is constructed and then the pre-defined kernel function can be calculated. The top 50 PCs of each spot serve as the beginning feature input and are aggregated with the guidance of the kernel function to obtain spots’ embeddings for spectral clustering to generate a cluster assignment and the intra-cluster distance is subsequently calculated during each iteration. When the intra-cluster distance starts to increase, iteration terminates and the cluster assignment in the iteration presenting the minimum intra-cluster distance is adopted as the pre-clustering result.

AGC works in a manner different from common GCN, as it generates embeddings every iteration for each spot through aggregating the feature of neighbors with a pre-defined non-trainable kernel, then spectral clustering is performed with the embeddings and correspondingly an intra-cluster distance is calculated. Once the intra-cluster distance in current iteration is lower than that in last iteration, it terminates iteration and presents the cluster assignment in last iteration as its final clustering result in the form of a matrix *C*_*0*_ ∈ *Z*^1×*s*^ (Fig 3).

DCF relies mainly on two kinds of convolution kernel of size 2×2 and 3×3, both of which have the dilation rate set to be 2. Two sub-frameworks, both of which consist of 4 blocks are harnessed in parallel and the first 3 blocks of each sub-framework are repetition of a 3-layer unit for 3 times (Fig 1B). The 3-layer unit comprises sequentially the dilated convolution layer (kernel size 2×2 and 3×3 respectively in the two sub-frameworks), a batch normalization layer and a ReLU activation layer (Fig 1B). The last blocks of the two sub-frameworks contain only a convolution layer with kernel size 1×1. The outputs of the two sub-frameworks are summed up by pre-specified weights to generate a feature image *O* ∈ *R*^*n*×*h*×*w*^, from which embedding matrix *E* ∈ *R*^*s*×*n*^ of all spots can be extracted according to the lattice coordinates (Fig 1B).

For one thing, a probability matrix is derived from *E* via softmax function to represent the probability of each spot being assigned to each cluster (Fig 1A). For another, the pre-clustering labels serve herein as pseudo-labels. The cross-entropy loss *L*_*p*_ can be computed with the probability matrix and the pseudo-labels (Fig 1A). The objective of the pre-training is to minimize the cross-entropy loss.

Besides the initialization of all trainable parameters with the values ascertained during the pre-training stage, the centroid representation matrix *μ* ∈ *R*^*n*×*n*^ is an extra trainable parameter declared in the stage, the first *n* refers to the cluster number and the second *n* demand the representation vector for each centroid should also be *n*-dimensional, thus enabling the computation of the distance between the embedding of each spot and the representation of each cluster centroid to get the probability distribution matrix *Q* ∈ *R*^*s*×*n*^ (Fig 1A). Another probability distribution *P* ∈ *R*^*s*×*n*^ can be computed from *Q* so that the minimization of KL divergence *L*_*1*_ between the two distributions form a part of the training objective. *P* is kept unchanged relatively to *Q* by updating the value of *P* matrix every 4 times of iterations. Besides, every spot is conferred to a label corresponding to the cluster with the highest probability according to the matrix of *Q*, whereby a cluster assignment *C* ∈ *Z*^1×*s*^ for all spots can be generated to provide pseudo-labels in each iteration. Pseudo-labels of high confidence can be picked out if the corresponding probability is higher than *q*_*cut*_ (here *q*_*cut*_ = 0.5). A cross entropy loss *L*_*2*_ can be calculated among spots with high confidence pseudo-labels and the corresponding probability (Fig 1A). Besides, the spatial continuity loss *L*_*3*_ is computed as the mean value of Euclidean distances between all pairs of closest neighboring spots in longitudinal, transverse and diagonal directions in the lattice based on spots’ embeddings in the matrix *E* (Fig 1A). The total loss *L*_*t*_ equals the weighted sum of *L*_*1*_, *L*_*2*_ and *L*_*3*_ (Fig 1A).

### Clustering of 12 DLPFC samples with STGIC

The 12 DLPFC samples can be divided into 3 groups by shape and cluster number, each group containing 4 samples. The samples with sample id from 151507 to 151510 consist of 7 clusters and nearly all the spatial domains according to annotated labels look like straight strip, those from 151669 to 151672 consist of 5 clusters and all the spatial domains present gentle wave shape, those from 151673 to 151676 comprise 7 clusters and all the spatial domains present obvious arcs.

**Fig 4:**
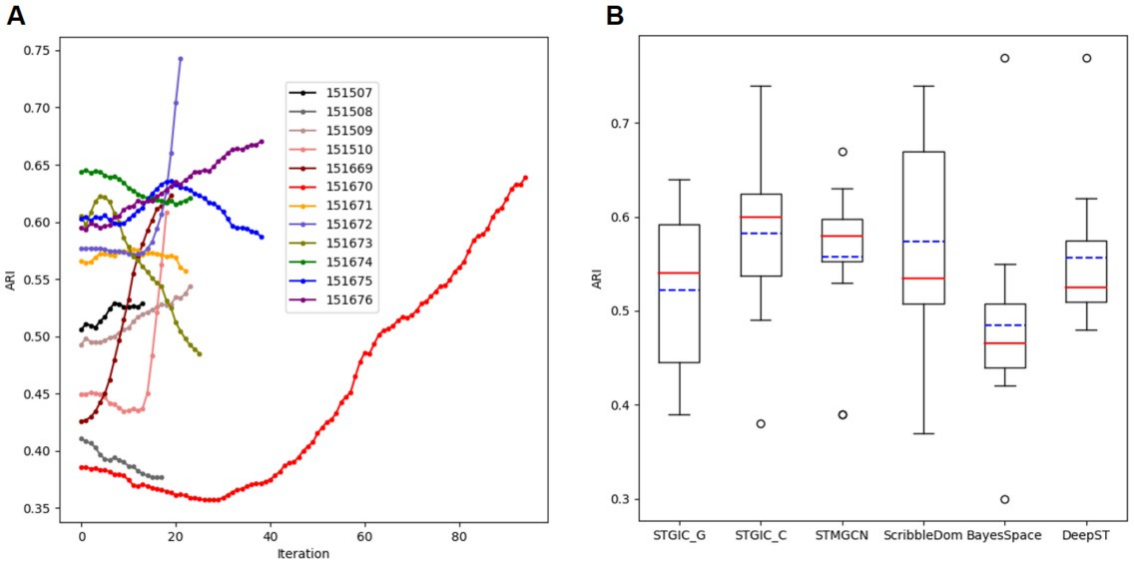
Variation trend of ARI measuring STGIC performance and comparison with baselines among the 12 DLPFCs. A. ARI curves with iterations going on for every sample. At the 0^th^ iteration, the ARI is calculated with the pre-clustering result and then is with the cluster assignment generated in each iteration of DCF training. B. Boxplot showing ARIs computed with clustering labels generated by STGIC and baseline methods. “STGIC_G” represents the pre-training of AGC in STGIC and “STGIC_C” represents DCF which presents the ultimate clustering labels for STGIC. The blue dash lines and red solid lines respectively mark the positions of mean and median ARI.

With a unified set of hyper-parameters during pre-processing, pre-clustering and clustering stages, the 12 DLPFC samples are clustered by STGIC with each slice once. Through clustering with DCF in STGIC, the mean and median ARIs are elevated to 0.58 and 0.60 from the level demonstrated by AGC pre-clustering. Although the general rising in performance is flawed by drops of ARI in several samples, it can be readily observed that the drops are so minuscule for most of the samples showing a lower ARI by clustering than by pre-clustering with only one striking exception of 151673 which demonstrates a significant ARI decrease (Fig 4A). For 151673, ARI has kept rising to 0.62 from 0.60 by the fourth iteration of DCF training, yet then goes through a continuous fall to 0.49 at the end, which may be imputed to the iteration termination mechanism adopted by STGIC, the function of DCF to extract feature, however, may still work. The trends of ARI curves for 151671 and 151675 unfold similar trends to 151673, but the drop of 0.01 is quite moderate. Only for 151508 and 151674, does STGIC not bring any ARI increases, but the drop remains within 0.03 (Fig 4A). One-tail paired t-test by the function stats.ttest_rel from the Python package of scipy validates the mean ARI of the benchmark dataset is significantly raised by DCF clustering from AGC pre-clustering and the p-value is 0.038.

AGC performs the best in the third group, but as far as this group is concerned, DCF tends to discount AGC’s achievement except in 151676. By contrast, in the remaining two groups which AGC is not very adept at, DCF tends to significantly improve the pre-clustering performance. The phenomenon suggests AGC and DCF are individually suitable for different kinds of samples and the combination of them guarantees a high performance overall.

**Fig 5:**
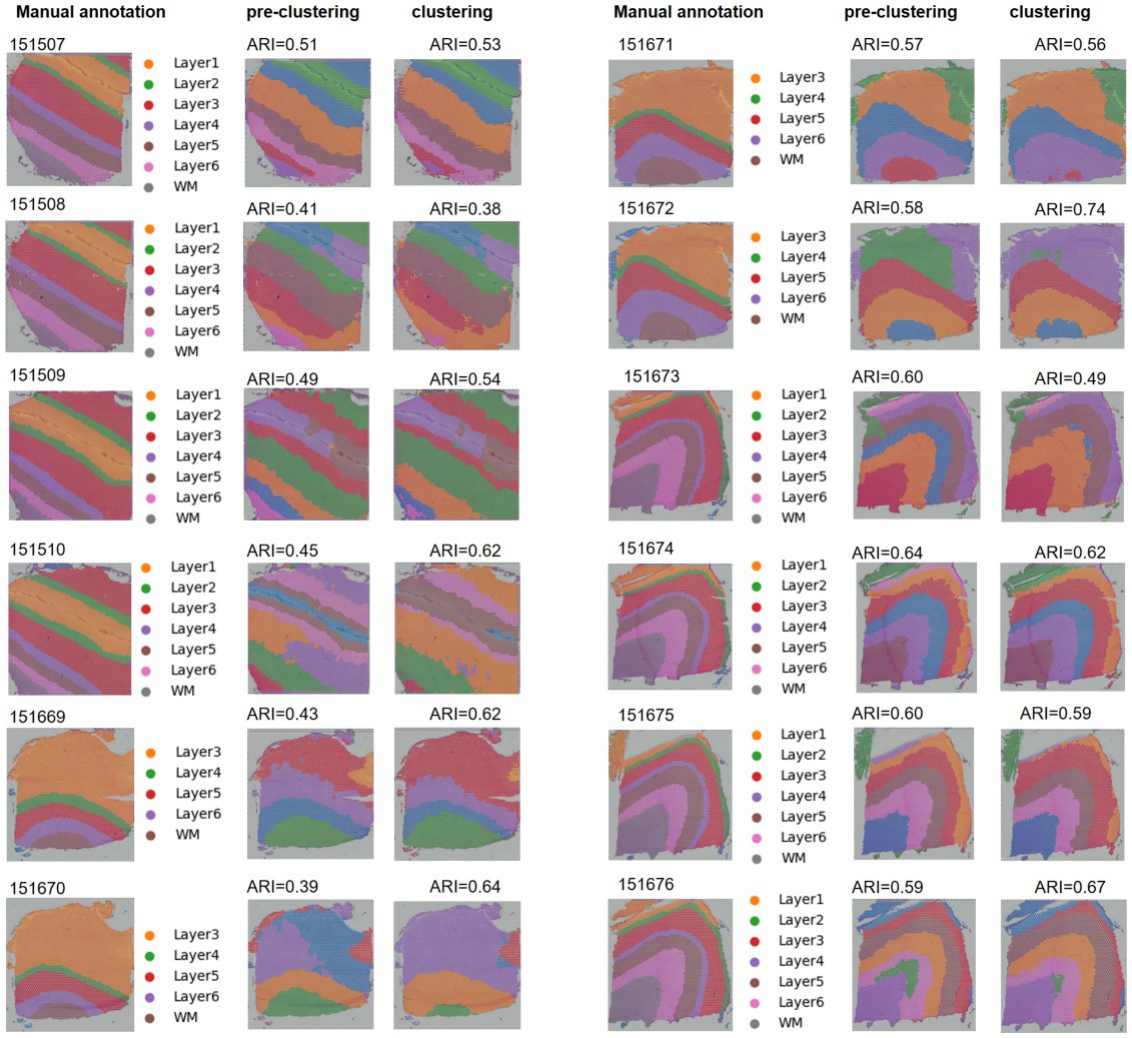
Annotated spatial domains as well as those derived from pre-clustering and clustering of STGIC. The figure legends about the labels are only for the annotated spatial domains.

Our pre-clustering and clustering results are compared with four baseline methods, including STMGCN, ScribbleDom, BayesSpace and DeepST. Three of them are unsupervised method different from ScribbleDom which depends on expert’s scribble annotations on a semi-supervised basis to attain SOTA performance. Although ScribbleDom can also be operated in an unsupervised way, the resulting performance will be discounted significantly. ScribbleDom as a baseline here is operated in the semi-supervised way. Among the baselines, the highest mean ARI is presented by the semi-supervised ScribbleDom to be 0.57, while the highest median is presented by STMGCN to be 0.58. The second highest mean ARI displayed by baselines is 0.56 which is reached by DeepST and STMGCN. BayesSpace falls far behind them in both mean and median ARI The mean and median ARI of AGC pre-clustering in STGIC are respectively 0.52 and 0.54, suggesting the pre-clustering AGC performs better than only BayesSpace. However, the mean and median ARIs 0.58 and 0.60 displayed by the final clustering of DCF in STGIC are the highest of all (Fig 4B), demonstrating the superiority in clustering accuracy of STGIC over all the baseline methods. The visualization of spatial domains in the 12 samples are depicted according to the pre-clustering and clustering labels generated by STGIC (Fig 5).

The above performance of DCF in STGIC on the benchmark dataset is attained with the input of the top 15 PCs generated by PCA with the top 3000 highly variable genes and with the learning rate of 0.01 at the training stage. Other numbers of principal components have also been tried with PCA done in the range of all genes and the top 3000 highly variable genes. Generally, numbers of principal components around 15 and 50 with PCA done in the range of top 3000 highly variable genes tend to lead to higher clustering performance than other cases when values of all other hyper-parameters are unchanged (see S1 Table). Besides the above highest performance of STGIC, 17 PCs derived from PCA with the top 3000 highly variable genes lead to both median and mean ARIs of 0.58 (see S1 Table), which are also not lower than the highest level of baselines. Furthermore, if the learning rate during the training stage drops to 0.005, the top 15 PCs derived from PCA with the top 3000 highly variable genes lead to both median and mean ARIs of 0.59. Compared with the learning rate of 0.01, the mean ARI is raised from 0.58 to 0.59, while the median ARI is decreased from 0.6 to 0.59. Given that comparisons in the clustering performance among the methods thereof are carried out more often in terms of the median ARI than the mean, the performance attained by the top 15 PCs resulting from the top 3000 highly variable genes with the learning rate of 0.01 is considered to be a little better than that achieved with the learning rate of 0.005 and hence the highest performance of STGIC. The group of hyper-parameter values leading to the highest performance is adopted in the analysis of all other 10x Visium data in the study.

In the analysis on non-benchmark datasets, STMGCN and DeepST will still be used to compare with STGIC, however, ScribbleDom and BayesSpace will no longer be considered in light of much lower clustering accuracy of BayesSpace than the other baselines and the dependence of ScribbleDom in performance on expert’s annotations which are not available for many non-benchmark datasets.

### Ablation study on DFC

**Fig 6:**
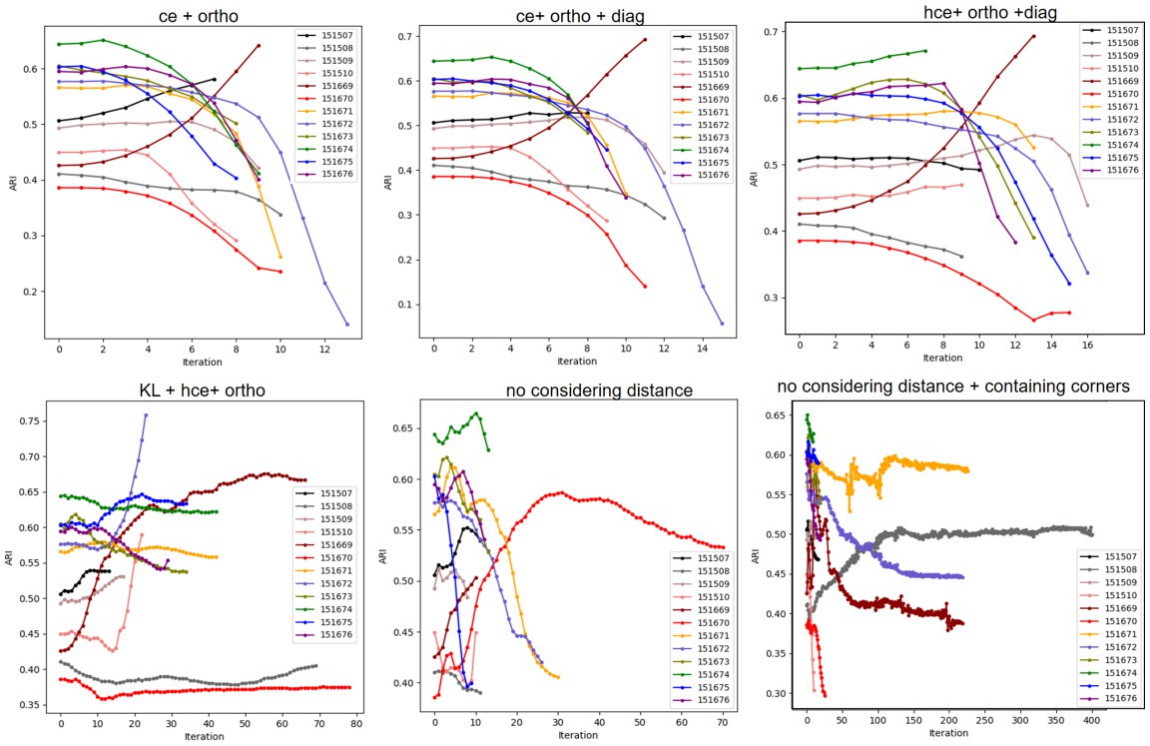
Variation trend of ARIs in all cases of ablation study.

To corroborate the necessity of adapting the training objectives adopted in the unsupervised image segmentation algorithm and TESLA, The KL divergence loss is removed and *q*_*cut*_ is set to be 0 so that all spots are involved in calculating cross entropy. Besides, the closest neighboring spots pairs in diagonal direction are not considered any more for calculating of spatial continuity loss, correspondingly, this kind of training objective is restored completely to those adopted by TESLA and denoted as “ce (cross entropy calculated with all spots) *+* ortho (spatial continuity in orthogonal directions)”. As a result, an awful performance is brought about with mean and median ARI demonstrated by DCF being 0.39 and 0.40. For individual samples, rising from pre-clustering ARI is only seen in 151507 and 151669 while the remaining samples suffer a slumping of ARI (Fig 6A). Restoration of spatial continuity loss in diagonal direction from the above situation doesn’t make any obvious difference (Fig 6B), the kind of training objective is denoted as “ce + ortho + diag (spatial continuity in diagonal direction)”. If *q*_*cut*_ is restored to 0.5, spots with high confidence pseudo-labels are screened for calculating cross entropy and spatial continuity loss in three directions are simultaneously calculated, the mode of training objective is denoted as “hce (cross entropy calculated with the spots which own cluster assignment of high confidence) + ortho + diag”, which raise the mean and median ARI to 0.45 and 0.41 (Fig 6C). The performance will be improved by a substantial extent if the self-supervision KL divergence loss is introduced to restore the integral training objectives of STGIC, as has been elucidated. However, to validate the positive effect of spatial continuity loss in the diagonal direction, the corresponding part is subtracted from the integral loss, and the mode of training objective is denoted as “KL + hce + ortho”, which demonstrates a performance a little worse than the integral objective does, since the mean and median ARI are both 0.56 (Fig 6D). Although this mode leads the clustering ARI in some samples to be even higher than the integral objective does, it fails in getting an ideal performance in some other samples, especially in the sample 151670. To validate the reasonability of the adaptation of convolution kernel weights to spatial distance, the related operation is abolished, decreasing the mean and median ARI to 0.49, and the mode is denoted as “no considering distance”. In the case, the clustering ARI is lower than the pre-clustering value in every sample except for 151669 and 151670 (Fig 6E). If the fixing of the weights at the 4 corners of the 3×3 kernel to be 0 is further canceled, the mean and median ARI decrease to 0.48 and 0.49, and the mode is denoted as “no considering distance + containing corners”. Among the 12 samples, only the ARI of 151508 rises after clustering (Fig 6F).

Incidentally, we have also tried with several contrastive learning designed for graph data and intended to transfer these methods to our problem as we deal with the self-supervision KL divergence. These contrastive learning methods including GCA [28], DGI [29] and GIC [30], don’t improve the performance significantly after integration into our existing training objective for DCF. Therefore, the framework of STGIC doesn’t take any of these contrastive learning skills.

### STGIC can resolve finer spatial domains in 10x Visium human breast cancer

**Fig 7:**
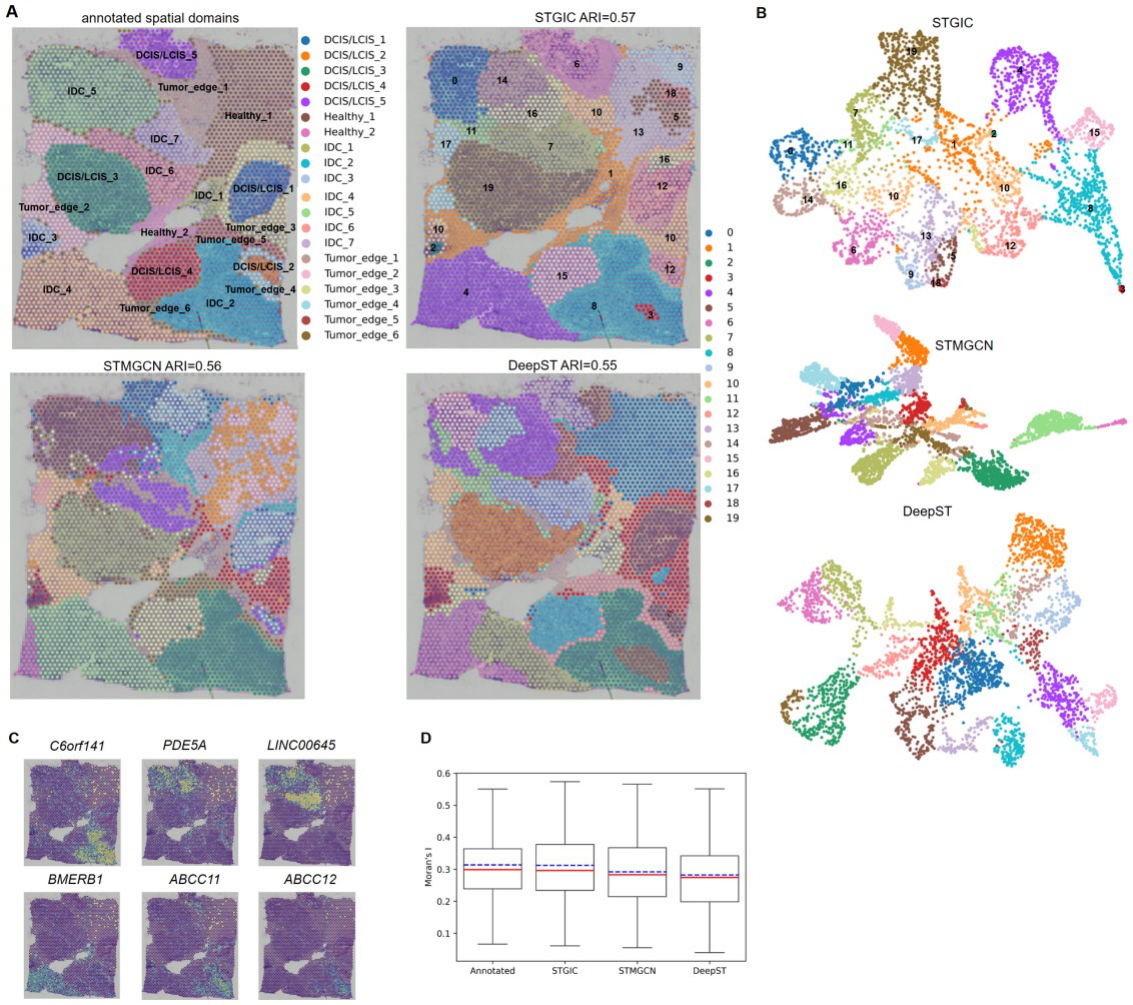
Analysis of 10x Visium human breast cancer based on STGIC clustering. A. Comparison of spatial domains identified by STGIC with those by the annotated labels and baselines. B. UMAP plot of spatial domains by clustering of STGIC and baselines. C. Spatial distribution of marker genes of invasive breast cancer. D. Boxplot of Moran’s I statistics of SVGs derived from the annotated labels, STGIC and baselines.

Manually annotated labels from SEDR’s author has been adopted herein to measure our pre-clustering performance with STGIC [31]. According to the annotated labels, the sample is divided into 20 spatial domains, which consist of 5 ductal carcinomas in situ (DCIS) or lobular carcinomas in situ (LCIS) regions with prefix “DCIS/LCIS”, 7 invasive ductal carcinoma (IDC) regions with prefix “IDC”, 6 tumor edge regions with prefix “Tumor_edge” and 2 healthy regions with prefix “Healthy”. Therefore, we pre-specify the cluster number to be 20 and use STGIC to cluster the sample, displaying an ARI of 0.57 measured with the reference to the annotated labels. ARI presented by STGIC with cluster numbers 18 and 22 are respectively 0.57 and 0.54 (see S1 Fig). Subsequent analysis on the dataset is done as to the cluster assignment generated at the cluster number prescribed by the ground truth. Our visualization of the resulting spatial domains demonstrates large overlaps with the main DCIS/LCIS and IDC regions, though some DCIS/LCIS and IDC regions are integrated into one domain in rare cases. Three small tumor edge regions with annotated labels “Tumor_edge_4”, “Tumor_edge_5” and “Tumor_edge_6” have no corresponding spatial domains as well as the smaller healthy region “Healthy_2” as they are contained in domains corresponding to other regions (Fig 7A). In UMAP [32] visualization, STGIC and DeepST display less compact layout of spots in the same clusters than STMGCN, however, they separate different clusters more widely apart (Fig 7B). The capability of STGIC is validated indirectly by expression of marker genes of invasive breast tumor in the predicted spatial domains (Fig 7C), which are *C6orf141*, *PDE5A*, *LINC00645*, *BMERB1*, *ABCC11*, *ABCC12* [21, 22, 33, 34]. *C6orf141* is detected as the top SVGs of STGIC-predicted domains 3 and 8, which join together to correspond to the annotated region “IDC_2”, and higher expression of the two drug-resistant genes *ABCC11* and *ABCC12* are also detected in these domains than in surroundings. *PDE5A* presents a high expression mainly in Domain 14 and 16 which are largely overlap with the region “IDC_5”. *LINC00645* is identified as the top SVGs of Both Domain 7 and 14, which together overlap largely with “IDC_5”, “IDC_6” and “IDC_7”. Domain 4 highly expressing *BMERB1* nearly coincides with the region “IDC_4” (Fig 7A and 7C). STGIC presents slightly higher ARI on the dataset than STMGCN and DeepST do (Fig 7A). Besides, Moran’s I statistics of SVGs detected by STGIC is slightly higher than that of SVGs detected by baselines and approaches the level of the ground truth very much (Fig 7D).

### STGIC can resolve finer spatial domains in 10x Visium mouse posterior brain

**Fig 8:**
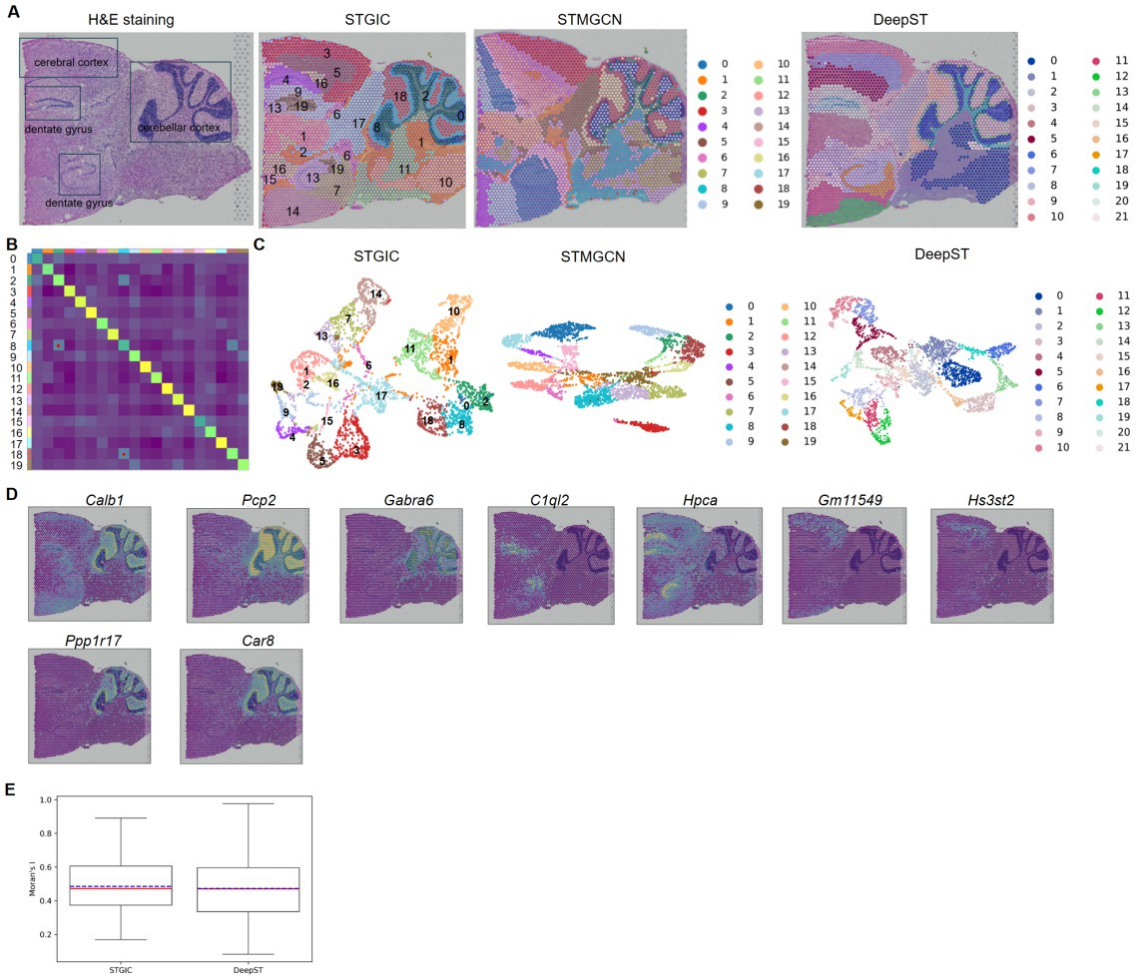
Analysis of 10x Visium mouse post-brain sagittal slice based on STGIC clustering. A. H&E histological slice image and visualization of Spatial domains identified by STGIC and baselines. B. Neighborhood enrichment plot of spatial domains identified by STGIC. Two pairs of spatial domains having the strongest neighborhood relationship are highlighted with red dot. C. UMAP plot of spatial domains by clustering of STGIC and baselines. D. Spatial distribution of marker genes corresponding to layers in cerebellar cortex, cerebral dentate gyrus and cortex. Marker genes highly expressed in the same domain identified by STGIC are arranged in the same column. E. Boxplot of Moran’s I statistics of SVGs detected by STGIC and the baseline.

STGIC manages to depict the fine-grained spatial domain of the sagittal slice of mouse posterior brain with the pre-specified cluster number of 20, especially in cerebellar cortex. In reference to the corresponding anatomical structure presented by Allen Mouse Brain Atlas, cerebellar cortex can be divided into three layers from outer to inner which are molecular layer, granular layer and fiber tract. These layers correspond respectively to our spatial domains 18, 8 and 2 (Fig 8A). The neighborhood relationship between spatial domains is calculated and visualized as a neighborhood enrichment plot which highlights spatial approaching among the above three domains (Fig 8B). *Pcp2*, *Car8*, *Calb1* and *Ppp1r17* are marker genes abundantly expressed in Purkinje cells in cerebellar cortex [16, 35, 36]. *Pcp2* and *Car8* are detected to be highly expressed in Domain 18, while *Calb1* and *Ppp1r17* are found to be top SVGs of Domain8 (Fig 8D). *Gabra6* [37] is marker genes highly expressed in granule neurons of cerebellum and detected as top SVGs of Domain 2 (Fig 8D). In addition to cerebellum, dentate gyrus in cerebrum is also depicted clearly by two of our spatial domains 13 and 19, in which *Hpca* and *C1ql2* [18,38], markers of dentate gyrus, are identified as their top SVGs (Fig 8D). The Cerebral cortex corresponds to two of our spatial domains 3 and 5, characterized respectively by the higher expression of the markers *Gm11549* and *Hs3st2* (Fig 8D), which are both marker genes of cerebral cortex [39,40]. The cluster number set to be around 20 is beneficial to delimiting the fine-grained sub-tissue structures, while the spatial domains demarcated with lower cluster numbers can only generate coarse depictions for STGIC and DeepST. STMGCN performs rather unsatisfactorily with various cluster numbers since it always generates obvious sector spatial domains which are not consistent with the real anatomic structure of brains evidently (Figs 8A and see S2). Different clusters are not so crowded together to overlap in the UMAP of STGIC and DeepST as in that of STMGCN (Fig 8C). Now that the spatial domains identified by STMGCN is not suitable for depicting the anatomical structure of posterior brain, the subsequent comparison is made between STGIC and DeepST herein. DeepST dissects the cerebellar cortex into three domains and the cerebral cortex into two domains as STGIC does, however, it fails to assign the same cluster label to the two dentate gyrus, which means it recognizes the two dentate gyrus as different tissue types. Although the Moran’s I statics of SVG sets derived by STGIC and DeepST are quite close, the two methods make obvious difference in Silhouette coefficient (SC) score and Davis-Bouldin (DB) score on the dataset. SC and DB generated by STGIC are 0.33 and 1.16, while those by DeepST are respectively 0.15 and 1.93. Generally speaking, the higher SC is, the better the clustering performance is, and the case for DB is in contrast. The two kinds of scores demonstrates STGIC generates a better spatial domain demarcation than DeepST on this dataset.

### Clustering derived from STGIC explicitly delineate the laminate structure of Stereo-seq mouse olfactory bulb

**Fig 9:**
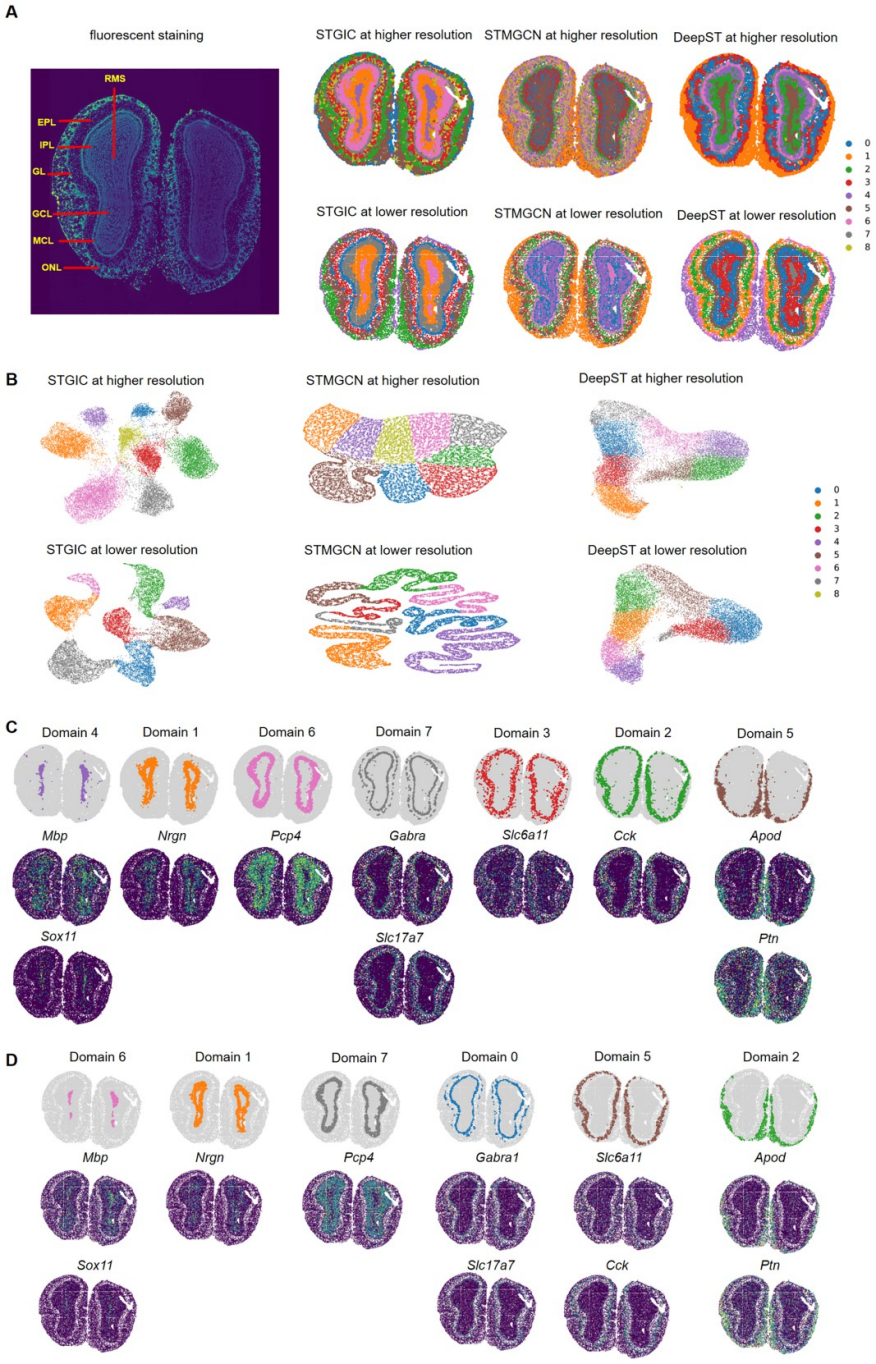
STGIC depicts layers of Stereo-seq mouse olfactory bulb with great clarity. A. Manually annotated layers of mouse olfactory bulb and visualization of spatial domains identified by STGIC and the baselines at cell level and lower resolution. B. UMAP plot of spatial domains by STGIC and the baselines at the two levels of resolution. C. Visualization of individual domains and spatial distribution of their marker genes for the cluster assignment as to data with cell-level resolution by STGIC. D. Visualization of individual domains and the spatial distribution of their marker genes for the cluster assignment as to data with lower resolution by STGIC. Marker genes are arranged in the same column as the corresponding domains in both C and D.

The Stereo-seq data is of high resolution at cellular level and has 19081 spots. Mouse olfactory bulb mainly consists of 7 layers which from the inner to the outer are RMS, GCL, IPL, MCL, EPL, GL and ONL (Fig 9A). These layers have been labeled in Allen Brain Atlas [41] and also by SEDR’s author [31]. Every layer has at least one marker gene. Visualizations of STGIC, STMGCN and DeepST predicted spatial domains with the cluster number 7 all fail to display clearly the innermost layer RMS (see S3 Fig). When the cluster number is set to be 9, each layer has its own corresponding spatial domain identified by STGIC. The Domain 4, 1, 6, 7, 3, 2, 5 are mapped to the above 7 layers in turn (Fig 9A) and marker genes of these layers all present much higher expression in the corresponding domains than in their neighboring domains (Fig 9C), which proves the precision of STGIC clustering indirectly. STMGCN and DeepST also demarcate the 7 typical layers at cluster numbers higher than 7. Pervasive overlaps occur to spatial domains identified by STMGCN, which blur the bounds seriously. DeepST presents the innermost three layers of RMS, GCL and IPL and the outmost layer of ONL clearly, but renders the middle layers vaguely. Therefore, for the dataset at high spatial resolution of cell level, STGIC depicts the laminate structure of the olfactory bulb more precisely than STMGCN and DeepST, and DeepST performs better than STMGCN. In light of the obvious blurring of spatial domains identified by STMGCN, SC and DB scores are compared only between STGIC and DeepST. STGIC presents a higher SC of 0.54 and lower DB of 0.72 than DeepST, those of which are respectively 0.41 and 1.86. The spatial domain visualizations and the two kinds of scores all validate the better clustering performance of STGIC. To further validate the superiority of STGIC, the resolution is decreased by binning the data to reserve 14781 spots. STGIC still manages to demarcates a majority of the anatomical layers except that EPL and GL are merged into Domain 5 (Fig 9A), as is demonstrated by the marker genes *Slc6a11* and *Cck* [18] of the two layers both presenting higher expression in this domain (Fig 9D). Since cells abundantly expressing the two genes are so close in spatial distribution as to be easily binned together, it is expected that a unified single domain displays high expression of the two genes. In general, for STGIC and the two baselines, the bounds between neighboring spatial domains are clearer at the lower spatial resolution which may be attributable to higher gene-capturing efficiency at the lower resolution. Higher gene-capturing efficiency ensures each spot can be represented by feature extracted from more genes such that the resulting feature input is able to describe the corresponding spot more precisely. At the lower spatial resolution, STGIC still presents clearer depiction of the 7 typical layers than STMGCN and DeepST. Diffusive distribution of spots belonging to same clusters remains rather obvious in the spatial domain visualization of STMGCN, which leads to a much poorer performance than STGIC and DeepST. At the lower resolution, the middle layers are depicted by DeeST more clearly than those by itself at the higher resolution. However, DeepST fails to demarcate the innermost two layers of RMS and GCL as clearly as STGIC does at the lower resolution. Besides, SC and DB of STGIC at the lower resolution are 0.66 and 0.48, while those of DeepST are respectively 0.48 and 1.52. At the two levels of resolution, STGIC separates different clusters in UMAP visualization more widely apart than STMGCN and DeepST (Fig 9B). All the above aspects support the superiority of STGIC to STMGCN and DeepST.

## Conclusions

We have developed a deep learning method STGIC for ST clustering. STGIC combines graph convolution AGC and an image convolution framework DCF together to attain high clustering performance on multiple datasets. It starts with AGC pre-clustering to obtain high quality pseudo-labels and takes advantage of special setup of convolution kernels in DCF to extract features from a virtual image derived from regular lattice on the chip of 10x Visium and Stereo-seq, making sure that all neighbor spots only in a certain distance are involved in updating the feature of each spot and contributions of these neighbors to the updated feature are correlated with spatial distance. The training of DCF doesn’t simply follow any existing method in ST clustering, but transfers the self-supervision skill originally for graph learning to our image field, the spatial continuity loss and pseudo-labels skill adopted by the forgoing unsupervised image segmentation algorithm are modified. The spatial continuity loss considers diagonal direction and the pseudo-labels skill is limited only to spots with high confidence pseudo-labels.

STGIC demonstrates SOTA precision on the benchmark of the DLPFC dataset consisting of 12 samples. Its value is also displayed by the capability of depicting fine structure of human breast cancer, mouse post-brain and olfactory bulb, the ability to guide the identification of marker genes through the predicted spatial domains as well as the expandability from 10x Visium to Slide-seq.

## Method

### Overview of datasets

All data analyzed in this paper are available in raw form from their original authors.

(1) The 10x Visium Human DLPFC dataset is available within the spatialLIBD package (http://spatial.libd.org/spatialLIBD). (2) The 10x Visium Human breast cancer dataset is downloaded from the website (https://support.10xgenomics.com/spatial-gene-expression/datasets). The manually annotated labels is recorded at website https://github.com/JinmiaoChenLab/SEDR_analyses/blob/master/data/BRCA1/metadata.tsv. (3) The 10x Visium MouseBrain dataset of sagittal slice is dowloaded from the website https://support.10xgenomics.com/spatial-gene-expression/datasets. (4) The processed Stereo-seq mouse olfactory bulb tissue having 19109 spots with cell-level resolution is downloaded from the website https://github.com/JinmiaoChenLab/SEDR_analyses. Then it is binned at two different levels of resolution respectively having 19081 and 14781 spots.

### Formal details of STGIC

#### A. Filtering conditions for 10x Visium and Stereo-seq

For both 10x Visium and Stereo-seq data, genes which are detected in less than 3 spots are filtered at the beginning of the preprocessing stage. Besides, for Stereo-seq data, spots are filtered in which less than 20 genes are detected.

#### B. Computation of Adjacency *A* for AGC input

Following the practice of SpaGCN to generate the self-looped adjacency from the distance matrix *D*, we derive the adjacency in the formula:

$$A_{ij}=e^{-\frac{d_{ij}^{2}}{2\sigma^{2}}}$$ (1)

*A*_*ij*_ is the element of the adjacency *A* in row *i* and column *j*, *d*_*ij*_ is the element of the distance matrix *D* in row *i* and column *j* which represents the Euclidean distance between the *i*^*th*^ and *j*^*th*^ spot calculated with the pixel coordinates, σ is a parameter identified by searching a series of values to make the mean of row sums of the difference between *A* and identify matrix equal a pre-specified hyper-parameter which is set to be 0.5 herein by default [16].

#### C. Conversion of transcription and spatial information to virtual image *X*

For 10x Visium data, *X* is generated by arranging each spot at the lattice coordinates rather than pixel coordinates since the spatial distribution would be too sparse for neighboring spots to be captured in one receptive field simultaneously if the pixel coordinates were adopted. The maximum abscissa and ordinate respectively adding 1 are the height and width of *X* as the coordinates start with 0 rather than 1. PCA is carried out as to the preprocessed gene transcription matrix *M* in the range of the top 3000 highly variable genes and the top 15 PCs are taken as pixel values of all spot pixels. The resulting 3000 dimensions of spots-averaged vector of the matrix *M* and the top 15 eigen-vectors of the above PCA are used to compute pixel values of the null and background pixels by subtracting the spots-averaged vector from a 3000-dimension zero vector and then computing the top 15 PCs with the mentioned top15 eigen-vectors. The imputation for background pixel is reasonable in that no cells exist in the background area and thus no expression of any genes, namely the gene expression is represented with zero vector. Our practice is to reduce zero vector to 15 dimensional with the eigen-vectors and average vector, which however seems inappropriate to process null pixels since their corresponding locations in the lattice are still in the area of tissue and could have had non-zero gene expression (Fig 2). Fortunately, this seemingly implausible point is circumvented by our introducing of dilated convolution kernel in DCF which ensuring the feature extraction of each spot pixel only refers to the neighboring spot pixels, eliminating the impact of the neighboring null pixels. Besides, only spot pixels are extracted from DCF-generated feature image to compute the value of loss function. Exclusion of background pixels and null pixels from loss calculation further attenuates the impact of the two kinds of unwanted pixels. For Stereo-seq data, only pixel coordinates are available, and hence *X* is constructed with pixel coordinates. Unlike the case in 10x Visium, the process will not cause sparse spatial distribution among spot pixels for the high resolution. Besides, closest spot pixels are not separated by null pixels because of the lattice structure. Therefore, we need only to compute pixel values for spot pixels and null pixels with the same method described above.

#### D. Obtaining of pre-clustering label *C*_*0*_ by AGC

The smoothed embedding matrix *H*_*t*_ ∈ *R*^*s*×*50*^ after iterations of *t* times from the initial feature matrix *F* is expressed as:

$$H_{t}={\left(I-L/\lambda \right)}^{t}F$$ (2)

*I* is identity matrix, *λ* is the approximate to the largest eigen-value of the symmetrically normalized Laplacian matrix *L*. we follow the practice of AGE to set *λ* to be 1.5 [42].

In each iteration, the smoothed embedding matrix multiplying its own transpose generates a similarity matrix, the eigen-vector matrix of which is used to carry out spectral clustering. Specifically, the top 26 eigen-vectors provide each of the *s* spots with a 26-dimension representation vector, based on which Clustering is performed with Kmeans to generate a cluster label assignment. The assignment *C*_*t*_ ∈ *Z*^*1*×*s*^ generated at the *t*^*th*^ iteration is used to calculate the intra-cluster distance *Intra*(*C*_*t*_) as:

IntraCt=1|Ct|∑l∈Ct1|l|(|l|-1)∑i,j∈li≠j||Hti-Htj||2 (3)

|*C*_*t*_| is the number of different cluster labels in *C*_*t*_, *l* is any of different labels in *C*_*t*_, |*l*| is the number of spots assigned to the label *l*, *i* and *j* are any pair of spots with different indices and assigned to the label *l*, *H*^*i*^_*t*_ and *H*^*j*^_*t*_ are respectively the smoothed embeddings of the *i*^*th*^ and *j*^*th*^ *spots* in the *t*^*th*^ iteration, || ||_2_ is L2-norm. Once the intra-cluster distance begins to increase, iteration will be terminated and cluster assignment obtained in the iteration immediately before the increase are taken as pre-clustering label *C*_*0*_.

#### E. Construction of DCF for feature extraction

**Fig 10:**
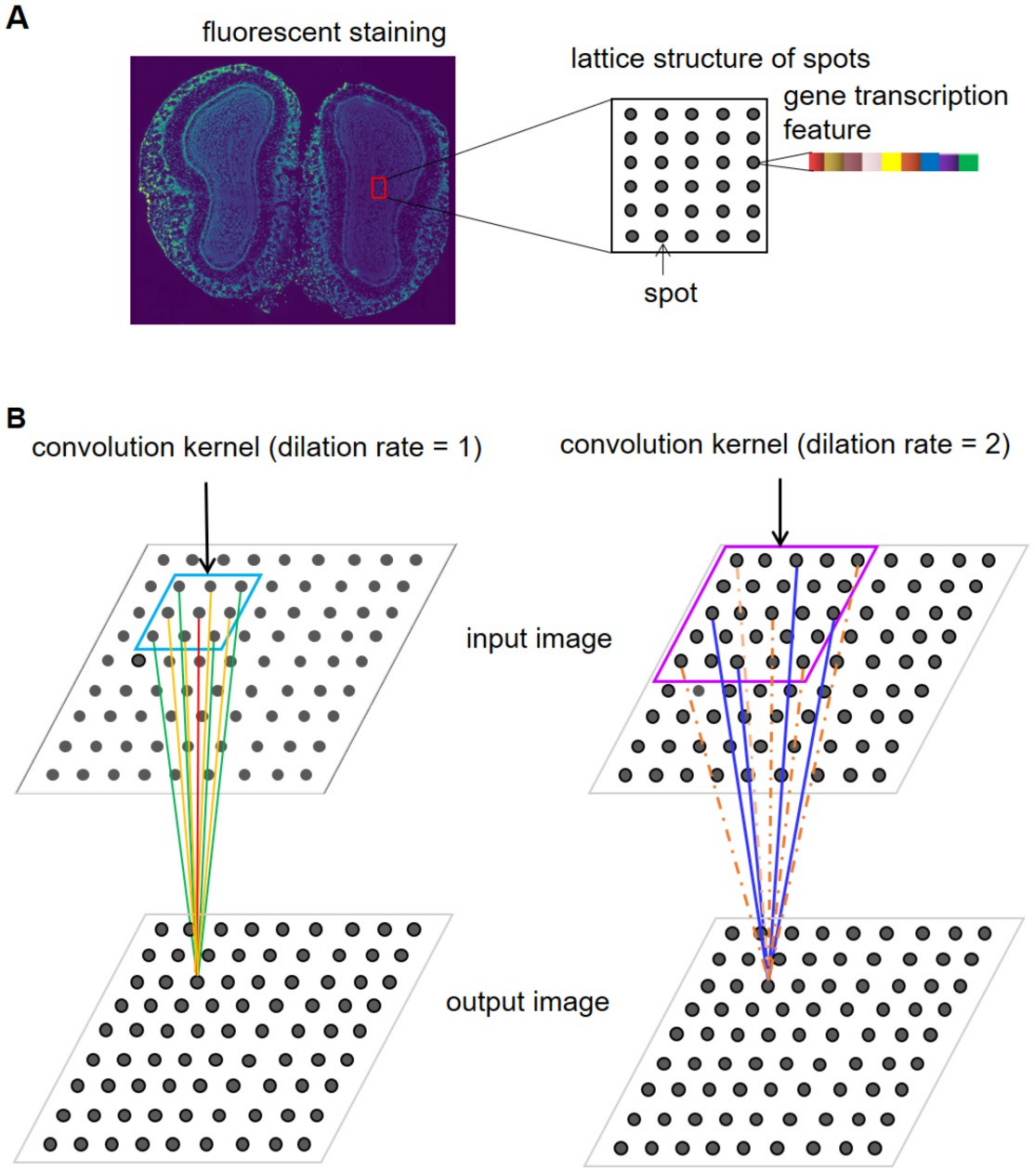
Lattice structure of Stereo-seq and the adaptation of convolution kernel to the structure. A. Spots are arranged one by one regularly in both longitudinal and transverse directions, quite different from what is adopted by 10x Visium on an alternate basis. B. Two kinds of convolution kernels are used in DCF, both have kernel size of 3×3, but one has dilation rate of 1 and the other has that of 2. Convolution kernel shares the same weight at positions with equal distance to the center. Lines with the same color represent the same kernel weight when extracting feature from a receptive field. Orange dash lines represent the weight of zero to ignore these spots during feature extraction.

Whether 10x Visium or Stereo-seq data is to be analyzed, DCF functions by its two sub-frameworks. As has been elucidated, the sub-frameworks contain two different kinds of convolution kernels. For 10x Visium data, the convolution kernels both have dilation rate of 2, but have different kernel sizes respectively of 3×3 and 2×2. The kernel weights at the four corners of the 3×3 convolution kernels are kept to be zero constantly to mask spots corresponding to the corners of receptive fields, since the spots in a receptive field acted upon by them are too far from the center to play an appreciable role in updating the feature of the center spot relatively to other spots in the same receptive field (Fig 1C). The enforcing of zeros as to the weights of all the corner elements of the 3×3 kernels is realized simply by setting the weights to be 0 regardless of how much they are after their updating by back-propagation. Besides, after updating the kernel weights through back-propagation during each iteration, adaptation of the kernel weights is implemented to the distance from the position of kernel elements to that of kernel centers such that same weight values are shared among elements equally far from the centers (Fig 1C). Specifically, the 2×2 kernels are adapted by declaring an extra trainable parameter, once all trainable parameters are updated by back-propagation, the value of the extra parameter is used to substitute those of the four corner elements, and the values of the elements at the centers and midpoints of edges of the 3×3 kernels are adapted in the similar way. The design of the combinatorial use of the two kinds of dilated convolution aims to ensure the calculation of the embedding of each spot pixel depends only on the 8 closest spot pixels and itself on a spatial distance correlated basis while precluding the interference of neighboring null pixels. The ultimate feature image is obtained by summing up the output feature images from the two sub-frameworks by certain weights which are set to be 0.7 for the one containing 3×3 kernels and 0.3 for the other. For Stereo-seq, it is noteworthy that spots are arranged continuously on the lattice so that spot pixels are distributed without any separation by null pixels in the virtual image *X* (Fig 10A). Correspondingly, we adjust the adaptation method of kernel weights to the characteristic of Stereo-seq. Convolution kernels with kernel size 3×3 and dilation rate respectively of 1 (no dilation at all) and 2 are respectively adopted in the two sub-frameworks. In addition to weights at the four corners of the dilated kernel with size 3×3 being kept zero all the way, the weight at the center should also be fixed at zero, since the center of receptive fields is paid attention to by the kernel without dilation and repetitive consideration of the center would otherwise overestimate its importance for updating the information of itself (Fig 10B). The operation guarantees the calculation of the embedding of each spot pixel involves only the 12 closest neighboring spot pixels together with itself in reference to the spatial distances. When the outputs from the two sub-frameworks are sum up to get the ultimate feature image, the pre-specified weight for the one from the sub-framework depending on kernels without dilation are set to be 0.9 and the other 0.1. For 10x Visum and Stereo-seq data, the feature images output by the first three blocks of all the sub-frameworks are set to have 100 channels and the feature image output by the last blocks of the sub-frameworks are set to have the channel number equal to the cluster number *n*. As is described above, the calculation of the embedding of each spot pixel in 10x Visium data involves less neighboring spot pixels than in Stereo-seq data. The setting is reasonable given that spots distribution on 10x Visium chips are sparser than those on Stereo-seq and the spatial distances between closest spots are larger in the former. The lower spot density on 10x Visium chips signifies fewer neighboring spots share transcriptional correlation with the centering spot.

#### F. Self-supervision training of DCF

A self-supervision trick realized via KL divergence firstly proposed by the community detection algorithm SDCN [43] and then employed by SpaGCN [15] is also used in our DCF. Given that the trick is as such designed for graph node embedding generated by GCN, we have to calculate with the *E* matrix composed of embedding of all spots extracted from the feature image of *O* instead of directly using it. The *Q* distribution is calculated as:

$$q_{ij}=\frac{{\left(1+{\left||E_{i}-\mu_{j}|\right|}_{2}^{2}\right)}^{-1}}{\sum_{j^{\prime }=1}^{\left|C\right|}{\left(1+{\left||E_{i}-\mu_{j\prime }|\right|}_{2}^{2}\right)}^{-1}}$$ (4)

*q*_*ij*_ is the probability of the *i*^*th*^ spot belonging to the *j*^*th*^ cluster, *E*_*i*_ is the embedding of the *i*^*th*^ spot in *i*^*th*^ row of the matrix *E*, *μ*_*j*_ is the representation vector of the *j*^*th*^ cluster centroid in the *j*^*th*^ row of the matrix *μ*, *|C|* is the number of different labels in the cluster assignment matrix *C*, || ||_2_ is L2-norm. Based on *Q*, the distribution *P* is calculated as:

$${\;p}_{ij}=\frac{q_{ij}^{2}/\sum_{i\prime }q_{i\prime j}}{\sum_{j\prime }\left(q_{ij^{\prime }}^{2}/\sum_{i\prime }q_{i\prime j\prime }\right)}$$ (5)

the subscripts have the same meaning with those in formula (4).

#### G. Learning rates for pre-training and training of DCF

For 10x Visium, learning rates during DCF pre-training and training are 0.05 and 0.01. For Stereo-seq, these two values are set to be 0.005 and 0.001.

#### H. Termination condition for pre-training and training of DCF

The maximum iteration number of pre-training and training are 200 and 400. Besides, the pre-training of DCF will stop if any of the following three cases occur: (*i*) the number of unique cluster labels generated at current iteration is less than the pre-specified cluster number *n*, (*ii*) at least one cluster exists in current iteration having the member spots account for less than a certain fraction (set to be 0.01 for the 12 DLPFC benchmark) of the total, (*iii*) current iteration experiences the updating of the matrix *P* and the ratio of spots having different cluster labels from those in last iteration to the total spots number *s* is lower than 0.001.

#### I. Weights of all parts of the loss for training DCF

The spatial continuity loss *L*_*3*_ is the weighted sum of two parts, the first is computed with the closest spots in the orthogonal (including longitudinal and transverse) direction and the second in the diagonal direction. Their weights are respectively 0.62 and 0.58 for 10x Visium, while they are 0.62 and 0 for Stereo-seq. The total loss *L*_*t*_ is the weighted sum of *L*_*1*_, *L*_*2*_ and *L*_*3*_ with their weights of 0.78, 0.71 and 1. Among the three components of losses, the weight ratio of *L*_*1*_ to *L*_*3*_ is critical to clustering accuracy.

### Identifying spatially variable genes

For STGIC-identified and annotated spatial domains, the python package SCANPY [44] is used to detect SVGs in each domain with the function scanpy.tl.rank_genes_groups implementing Wilcoxon test. Differential expression of genes in each spatial domain is investigated with the cutoff of adjusted p-value 0.05 and foldchange of 1.5. Besides, the identification of SVGs depends on two additional criteria: (1) the percentage of spots expressing the gene in the target domain, that is, in-fraction, is >80%; (2) for each neighboring domain, the ratio of the percentages of spots expressing the gene in the target domain and the neighboring domain(s), that is, in/out fraction ratio, is >1. The above criteria are taken from SpaGCN [16].

### Neighborhood enrichment of spatial domains

The function of squidpy.gr.nhood_enrichment from the python package SQUIDPY [45] is used to calculate the extent to which any two spatial domains are neighboring to each other.

### Calculation of spatial autocorrelation of gene expression

The autocorrelation is measured by Moran’s I score and implemented with the function squidpy.gr.spatial_autocorr from the package SQUIDPY.

### Calculation of clustering performance

When annotated labels is provided, clustering performance can be computed with the annotated and predicted labels. Suppose *U* = {*U*_*1*_, *U*_*2*_,…, *U*_*cn*_} are the set of annotated labels containing *c*_*n*_ different elements and *V* = {*V*_*1*_, *V*_*2*_,…, *V*_*cp*_} are the set of predicted labels containing *c*_*p*_ different elements, clustering performance is measured by ARI, which is calculated as:

ARI=∑i,jnij2-∑ini.2∑jn.j2n212∑ini.2+∑jn.j2-∑ini.2∑jn.j2n2 (6)

*n*_*i*._ and *n*_.*j*_ are the number of spots categorized as *U*_*i*_ and *V*_*j*_. *n*_*ij*_ is the number of spots simultaneously attached to *U*_*i*_ and *V*_*j*_. The higher ARI score is, the more consistent the predicted labels are with the annotated ones.

When ground truth cluster label is absent, embeddings and the corresponding predicted cluster labels can be used to calculate Silhouette Coefficient (SC) score and Davis-Bouldin (DB) score with the functions in the Python package of sklearn. SC with the range from -1 to 1 is related to the mean intra-cluster distance and the mean nearest-cluster distance, the higher the score demonstrates the better clustering performance. DB is the average similarity of each cluster with its most similar cluster. The similarity is measured by the ratio between intra-cluster distance to inter-cluster distance. The minimum of DB is 0, and the lower score demonstrates better clustering [21].

### Package version

All the software and packages are listed in Table 1:

**Table 1:** Version of software and packages involved in STGIC.

Package	Version
Python	3.8.13
Pytorch	1.13.0+cu117
numpy	1.20.1
pandas	1.2.1
scikit-learn	0.23.2
scanpy	1.9.1
skmisc	0.1.4
scipy	1.6.2

### System and GPU

All experiments are executed on a Centos7.9.2009 server equipped with an NVIDIA A100 GPU (NVIDIA-SMI 530.30.02).

## Supporting information

S1 File Supporting information. S1 Text: Rationale of Graph Convolution Neural Network (GCN) and the Adaptive Graph Convolution (AGC) in STGIC.
(DOCX)

S1 Fig Comparison of STGIC with baselines on the human breast cancer data with 18 and 22 clusters. A. Visualization of Spatial domains with cluster number 18 and the corresponding UMAP plot. B. Visualization of spatial domains with cluster number 22 and the corresponding UMAP plot.
(TIF)

S2 Fig Visualization of spatial domains identified by STGIC and baselines on the mouse posterior brain data. The plot in the first row results from cluster number 10 and that in the second row from cluster number 15.
(TIF)

S3 Fig Spatial domains visualization with the cluster number 7 on the mouse olfactory bulb data. The plot in the first row results from STGIC, STMGCN and DeepST respectively at the higher spatial resolution and the second row from those at the lower spatial resolution.
(TIF)

S1 Table Mean and median ARI presented by STGIC on the DLPFCs dataset with various numbers of principal components around 15 and 50. n_components: number of principal components; all_mean: mean ARI resulting from PCA with all genes; all_median: median ARI resulting from PCA with all genes; hvg_mean: mean ARI resulting from PCA with the top 3000 highly variable genes; hvg_median: median ARI resulting from PCA with the top 3000 highly variable genes.
(DOCX)
