# Supplementary material for: STGIC: A graph and image convolution-based method for spatial transcriptomic clustering
Source: PLoS Comput Biol. 2024 Feb 28;20(2):e1011935. doi: 10.1371/journal.pcbi.1011935 (PMC10927115; doi:10.1371/journal.pcbi.1011935)
Supplement: S1 File — S1 Text: Rationale of Graph Convolution Neural Network (GCN) and the Adaptive Graph Convolution (AGC) in STGIC. (DOCX) [file pcbi.1011935.s001.docx]

**STGIC: a graph and image convolution-based method for spatial transcriptomic clustering**

**S1 Text: Rationale of graph convolution neural network (GCN) and the adaptive graph convolution (AGC) in STGIC**

For a graph with n nodes, the adjacency matrix *A* and the degree matrix *D* are used to compute the Laplacian matrix *L*=*D*-*A*, which is then symmetrically normalized by *D^-1/2^* to get *L_s_* = *I* - *D^-1/2^AD^-1/2^* [1]. The normalized Laplacian matrix can be eigen-decomposed in the form of *UΛU^-1^*, *Λ* is the diagonal matrix with the eigen-values *λ_1_* ≥ *λ_2_* ≥ ... ≥ *λ_q_* ≥ ... ≥ *λ_n_* at the diagonal, among which *λ_1_* is the largest eigen-value and also denoted as *λ_max_*. These eigen-values represent the frequencies of the graph, *U* is the corresponding eigen-matrix. All columns of *U^-1^* constitute a group of bases for graph Fourier transformation while those of *U* form bases for the inverse transformation. Every column vector of the feature matrix *X* provides a graph signal *f* in the spatial field with the information of all nodes. Fourier transformation of *f* is realized by left multiplication of it with *U^-1^*. In most cases, the graph convolution kernel is not predefined and contains trainable parameters. Fourier transformation of a graph convolution kernel can be abstractly denoted as a column vector (*g*(*λ_1_*), *g*(*λ_2_*),..., *g*(*λ_q_*),...,*g*(*λ_n_*))^T^, where *g* is a function of the eigen-values. After arranging the elements of the column vector in diagnonal and constructing a diagonal matrix denoted as *g(Λ)*, the Hadmard product between the two column vectors resulting respectively from Fourier transformation of the kernel and signal *f* is converted to the multiplication between matrix *g(Λ)* and *U^-1^f*. According to Convolution Theorem that Fourier transformation of convolution between two functions amounts to the product of Fourier transformation of the two functions and the lemma that the inverse process of a Fourier transformation of a function return to the function itself, the filtered signal f’ after the graph convolution operation can be calculated as:

$f^{'}=Ug(\Lambda)U^{-1}f$ (1)

which can further be expressed in a more compact form as *g(L_s_)f*. To construct GCN, Chebyshev polynomial which demands the independent variable fall into the range of the interval [-1,1] is often used to approximate *g*(*L_s_*) [1]. The restriction on the range of the independent variable is satisfied by transformation of *Λ* to 2*Λ/λ_max_ - I* and then *g*(*L_s_*) can be approximated with the following formula:

$g\left( L_{s} \right)=\sum_{k=0}^{K-1} w_{k}T_{k}(\frac{2L_{s}}{\lambda_{max}}-I)$ (2)

*K* is the order of Chebyshev polynomial, while *w_k_* is Chebyshev polynomial coefficient corresponding to the order *k*. *T_0_(X)=I*, *T_1_(X)=X*, and the recursion formula is

$T_{k}\left( X \right)=2XT_{k-1}\left( X \right)-T_{k-2}(X)$ (3)

GCN often takes these Chebyshev polynomial coefficients as trainable parameters whose values are identified on an iterative basis, rather than adopt a pre-defined graph convolution kernel function to compute these coefficients directly. If we set *L’=2L_s_/λ_max_ – I,* the *j^th^* column of output matrix of a GCN can be computed with the formula:

$f_{j}^{'}=\sum_{k=0}^{K-1} (T_{k}(L')\sum_{i=1}^{D_{in}} w_{kij}f_{i})$ (4)

where *D_in_* is input dimension, *f_i_* is the *i^th^* column of input matrix *X* and *w_kij_* is the corresponding Chebyshev polynomial coefficient to be trained. The output of GCN can be computed in the formula:

$F_{out}=\sum_{k=0}^{K-1} T_{k}(L')F_{in}W_{k}$ (5)

where *F_in_* is the input feature matrix, and *W_k_* is the trainable parameter matrix of the *k^th^* order Chebyshev polynomial coefficients [1].

GCN works well in supervised and semi-supervised learning where at least a part of ground-truth labels is available for training [2]. However, in clustering task where the ground-truth labels are thoroughly absent, it would be impossible to identify the trainable Chebyshev polynomial coefficients if self-supervision or contrast learning skills were not used to construct training objectives. Under the circumstance, graph convolution with pre-defined kernel function can be resorted to. The adaptive graph convolution AGC [3] for pre-clustering in STGIC works in this way to smooth spots’ embedding moderately. The underlying principle would be very clear if signal *f* in spatial field is decomposed by inverse Fourier transformation into *UZ*, where *Z* is a column vector comprising the Fourier coefficients as its elements corresponding to the columns of *U*, namely, the inverse Fourier bases. Substituting *f* with the decomposed form in formula (1) leads to the following formula to compute the filtered signal *f’*:

$f^{'}=\sum_{q=1}^{n} g(\lambda_{q})z_{q}u_{q}$ (6)

where *z_q_*_,_ the *q^th^* element of *Z* is the weight of the inverse Fourier base *u_q_*. Obviously, *g*(*λ_q_*)*z_q_* is the Fourier coefficient of the inverse Fourier base *u_q_*. Decreasing the Fourier coefficient of high frequency-corresponding basis drops the proportion of high frequency components and is more likely to generate a smoother filtered signal *f’*. Therefore, adopting a decreasing and non-negative function in the interval [0, *λ_max_*] for *g* meets the need of achieving ideal smoothness [3]. AGC pre-defines the Fourier-transformed convolution kernel function as follows:

$g\left( \lambda_{q} \right)=1-\lambda_{q}/\lambda_{max}$ (7)

and *f’* can be calculated with the formula:

$f^{'}=\left( I-L_{s}/\lambda_{max} \right)f$ (8)

After certain times of operation with the predefined kernel function, the resulting filtered signal *f’* is smoothed enough, the output embedding *E_s_* from the initial feature matrix *X* through *t* times of the above smoothing operation is computed in the formula:

$E_{s}={(I-L_{s}/\lambda_{max})}^{t}X$ (9)

**References**

1. Defferrard M, Bresson X, Vandergheynst P. Convolutional Neural Networks on Graphs with Fast Localized Spectral Filtering. arXiv preprint arXiv: 160609375v3. 2017.

2. Jiang B, Zhang Z, Lin D, Tang J, Luo B, editors. SEMI-SUPERVISED CLASSIFICATION WITH GRAPH CONVOLUTIONAL NETWORKS. 2019 IEEE/CVF Conference on Computer Vision and Pattern Recognition (CVPR).

3. Zhang X, Liu H, Li Q, Wu X-M. Attributed graph clustering via adaptive graph convolution. arXiv preprint arXiv: 190601210v1. 2019.
