# Supplementary figures and images for: STGIC: A graph and image convolution-based method for spatial transcriptomic clustering
Source: PLoS Comput Biol. 2024 Feb 28;20(2):e1011935. doi: 10.1371/journal.pcbi.1011935 (PMC10927115; doi:10.1371/journal.pcbi.1011935)

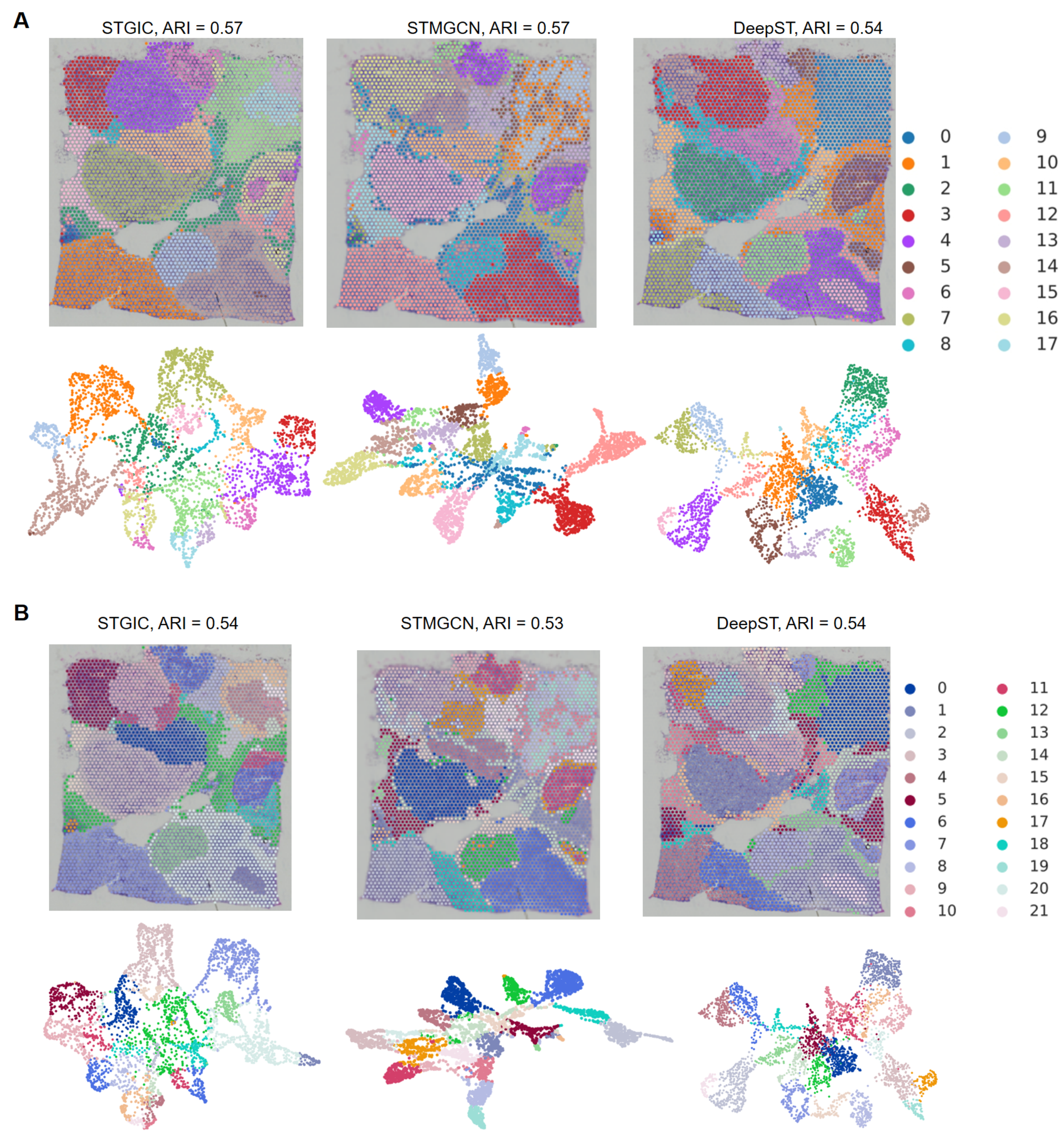

Supplement: S1 Fig — A. Visualization of Spatial domains with cluster number 18 and the corresponding UMAP plot. B. Visualization of spatial domains with cluster number 22 and the corresponding UMAP plot. (TIF) [file pcbi.1011935.s002.tif]

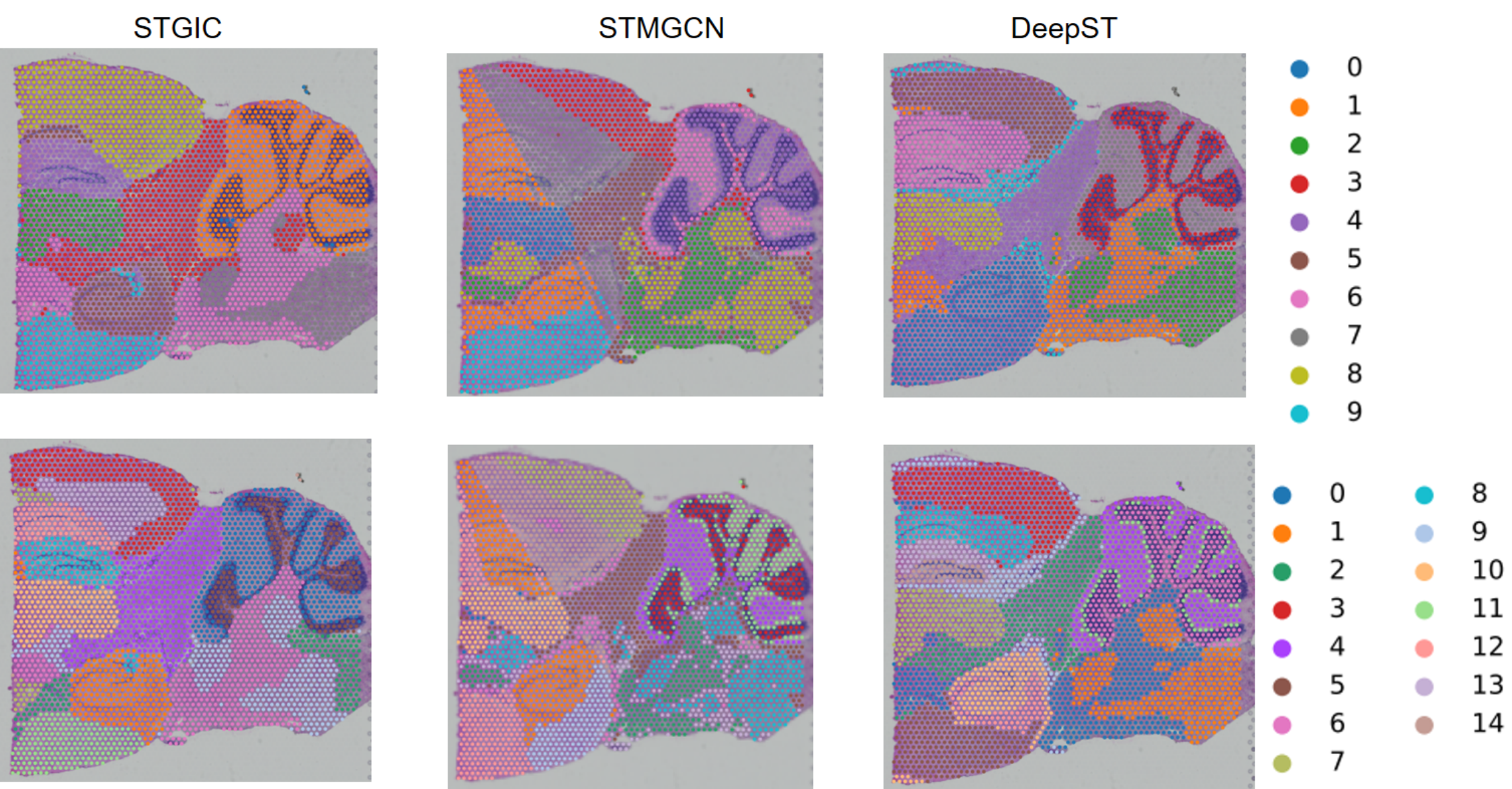

Supplement: S2 Fig — The plot in the first row results from cluster number 10 and that in the second row from cluster number 15. (TIF) [file pcbi.1011935.s003.tif]

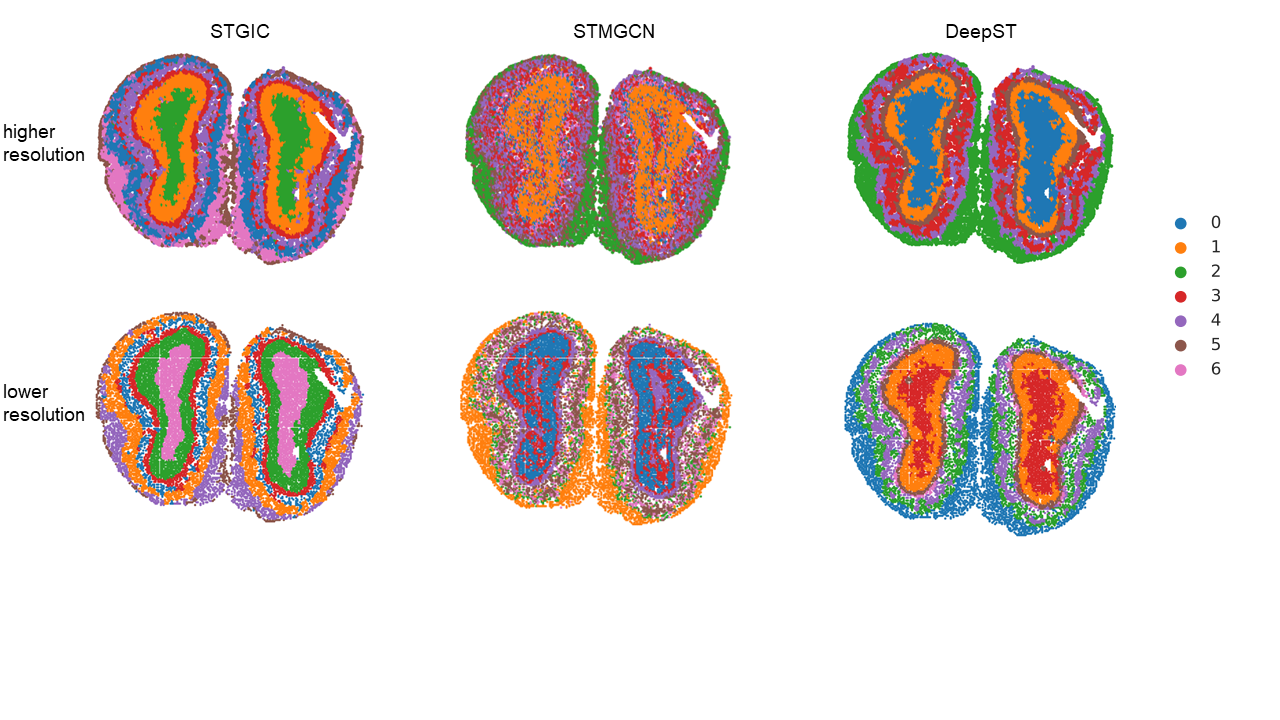

Supplement: S3 Fig — The plot in the first row results from STGIC, STMGCN and DeepST respectively at the higher spatial resolution and the second row from those at the lower spatial resolution. (TIF) [file pcbi.1011935.s004.tif]
