# Supplementary material for: STGIC: A graph and image convolution-based method for spatial transcriptomic clustering
Source: PLoS Comput Biol. 2024 Feb 28;20(2):e1011935. doi: 10.1371/journal.pcbi.1011935 (PMC10927115; doi:10.1371/journal.pcbi.1011935)
Supplement: S1 Table — n_components: number of principal components; all_mean: mean ARI resulting from PCA with all genes; all_median: median ARI resulting from PCA with all genes; hvg_mean: mean ARI resulting from PCA with the top 3000 highly variable genes; hvg_median: median ARI resulting from PCA with the top 3000 highly variable genes. (DOCX) [file pcbi.1011935.s005.docx]

**S1 Table. Mean and median ARI presented by STGIC on the DLPFCs dataset with various numbers of principal components around 15 and 50.** n_components: number of principal components; all_mean: mean ARI resulting from PCA with all genes; all_median: median ARI resulting from PCA with all genes; hvg_mean: mean ARI resulting from PCA with the top 3000 highly variable genes; hvg_median: median ARI resulting from PCA with the top 3000 highly variable genes.

| **n_components** | **all_mean** | **all_median** | **hvg_mean** | **hvg_median** |
| --- | --- | --- | --- | --- |
| 15 | 0.54 | 0.53 | 0.58 | 0.60 |
| 17 | 0.52 | 0.50 | 0.58 | 0.58 |
| 46 | 0.54 | 0.52 | 0.56 | 0.58 |
| 48 | 0.54 | 0.53 | 0.57 | 0.56 |
| 50 | 0.56 | 0.56 | 0.57 | 0.57 |
